# Analectic

**Published:** 1835

**Authors:** 


### MISCELLANEOUS INTELLIGENCE.

### ANALECTIC, ANALYTICAL, AND ORIGINAL.

### I.—ANALECTIC,

*Ad Sylvas Nuncius.*

ART. 1. *Bulletin Generale de Therapeutique Medicale et Chirurgicale, of May*, 1835.—(Some reflections upon the Medication entitled Diuretic.) The author of this paper goes into a short discussion of the reasons of the immense richness of the materia medica, and the real poverty of the therapeutics which have been bequeathed to us. He attributes it, in a great measure to the preceding age, having been too much in the habit of watching every new phenomenon in the operation of a prescribed substance, and setting it down as a specific for that effect. This produced, at the commencement of the present age, a re-action; and the study of the physiology and anatomy of patients and diseases was carried so far, that we almost forgot how to treat them. This view is strikingly applicable to the class of specific diuretics.

We understand by a diuretic, a medicine, which, when introduced into the economy, manifests such properties, that, under their influence, the subject voids a greater quantity of urine than ordinary. With so vague a definition, we have a vast number of diuretics, which are only so relatively; depending on the condition of the body with regard to the state of the pulmonary, cutaneous and intestinal exhalations; whether an increased secretion of urine is not necessary to maintain the equilibrium of the functions, etc. In order to ascertain whether a substance be a diuretic or not, we must compare the quantity of urine with the liquids taken into the digestive organs; we must ascertain exactly the state of the organism at the moment of the excreture, and we must compare the quantity of the urine with that of the water drank.

We should not place under this head, medicines which change a morbid condition of the organism, in which, by the simple fact of its cure or relief, it happens that the patient will discharge, by an abundant watery secretion, a liquid with which his tissues or his cavities have been filled. Thus, in cases of long continued intermittent fever, where the spleen has been enlarged or hardened, the tissues all appear infiltrated, and the peritoneum filled with serum, when treated with sulphate of quinine in large doses, (20 to 40 grains a day,) they lose the excessive volume of the spleen, the returns of fever, the general œdema, and the swelling of the abdomen, at the same time that large quantities of urine are voided. The sul. quinine is the diuretic here.

Likewise in cases of general infiltration, resulting from diseases of the heart, in which digitalis and repose have been prescribed, as the heart returns to its proper action, and the circulation resumes its normal rhythme, urine in large quantities is voided, and the œdema disappears. Repose and digitalis are here the duretics.

In the convalesence of long and debilitating disorders, when the patient begins to resume the sitting or standing posture, the inferior extremities become infiltrated in a greater or less degree, and it is only by degrees, as the strength returns in consequence of the regimen followed, and the tonic remedies prescribed, that the œdema disappears after some days of diuresis. The tonics employed to hasten and confirm the convalesence in this case, remove the fluid.

It sometimes happens, that subjects weakened by passing nights in the rain and cold, and exposed to the inclemencies of the seasons, are rapidly swelled up by a general leucophlegmacy. As this is not the result of an organic alteration, a few days of confinement to the bed and warm clothing are sufficient to cure it, and remove the œdema, frequently by the urinary passages.

The internal administration of opium, or the application of the salts of morphine, either internal or applied to the dermoid covering, often produces a retention of urine. As soon as the exhibition of them ceased, the pain and distress felt at the neck of the bladder is relieved, and the patient, then urinating abundantly and freely, seems as if a true diuretic had been administered to him.

In cases of irritation of the kidneys, whether produced by a calculus or otherwise, or by sympathy with an irritated bladder, we know that the best diuretic consists in calming the sympathetic or local irritation of the kidneys; since, under the influence of these means, the urine returns more natural and more abundant.

Finally, in almost all serious affections, do we not see the diminished, thickened and suppressed urine, reappearing more and more natural and abundant, under the influence of the treatment which relieves the principal disorder.

By thus rigorously distinguishing between diuresis, relative and direct, we shall be able to class those substances as diuretics which are properly such; and it is only in this way, that we can relieve ourselves of the ancient pharmacological chaos, or the modern therapeutic nullity.— *U. S. Medical and Surgical Journal, November*, 1835.

2. *On the Theraputic employment of Vapor baths in certain ocular Affections.*— The utility of vapor baths, either simple or aromatic, in a great variety of ocular diseases, although recommended and practiced by several distinguished physicians, is not sufficiently appreciated by the medical and general public.

This, doubtless arises, in many cases, from the difficulty of procuring an apparatus adapted to the purpose, especially in villages and country places. The Russians make a vapor bath by projecting a fine stream of water upon a heated stone placed in a close room, by which means the body is rapidly enveloped in steam. This is a very common custom with the common classes there, and the wives of these classes, who, during their pregnancy and puerperal state, take these baths enjoy much better health than those of the upper classes, who daily forsake the old national customs. Purulent ophthalmia is much less frequent with the new-born infants of the poorer than of the richer class. Who will deny, that of 100 cases of purulent ophthalmia in new-born infants, 95, at least, are attributable to a catarrhal inflammation of the palpebral and ocular conjunctiva? Is not this affection as much the result of a general refrigeration of the dermoid covering, as of the impression of the cool air upon the organ of vision?

In Egypt, the newly arrived European, when his conjunctiva is observed to be injected, and to put on the catarrhal form of the Egyptian ophthalmia, so rapid and so cruel in its results, is greeted with the exclamation, "How do you sweat?"

In incipient catarrhal ophthalmia of children, inquiry should always be made by the physicians as to the state of the skin: and if it is dry, cold and rough, it should be exposed to the vapor of emollient and sudorific plants: it should be enveloped in flannels, impregnated with vapor; and as the skin resumes its functions, the conjunctival coryza, which rapidly degenerates into an inflammation, with such a frequent sorrowful termination, will begin to disperse.

To an inflamed lachrymal tumor, an inflammatory tumefaction of the eyelids, either idiopathic, or following variola, or any other exanthematous affection, no more rational application can be made than emollient, resolutive, calming vapor bathings. In rheumatic or gouty sclerotic affections, vapor baths, applied to half the body, are found eminently useful. These may be obtained by putting the body in a cask, and filling it with vapor, after the Russian mode. This constitutes an excellent revulsive, which may at will be converted into a powerful derivative, by allowing it to proceed, in the lower extremities, to vesication, which is as active as, and ten times less painful, than the dry vesication.

The author of this paper has often employed calming and narcotic vapors in convulsive and cancerous affections of the eyelids and of the eyes with success. The leaves of the *laura-cerasus*, dried and cut up, and those of the *datura stramonium*, were the principal assistants. There are a great number of specific ocular affections, resisting ordinary treatment, which are cured by dry fumigations. Such are syphilitic ophthalmias, iritis of the same nature, exostoses, and tumors of the orbit, which are easily resolved under the employment of the vapor of the mercurial powders of Lalonette, of cinabar, or of the black sulphuret of mercury. Arsenious fumigations, directed to the cancerous pimples of the eyelids, often appease violent pain. Three cases are related, to show the rapidity which obstinate and long standing ocular affections are relieved by these means.

3. *Of the actual state of the Theraputics concerning the more serious contractions of the urethra.*—Up to the time of John Hunter, all the means used for the removal of this disorder may be reduced to simple *gradual dilitation;* for the treatment of the ancients with bougies, possessing various balsamic properties, amounts to nothing more. *Cauterization*, for some years, made considerable noise; but it has now lost much of its reputation. Caustics frequently prove worse than bad. A solution of *lapis infernalis*, of two grains to the onnce of distilled water, injected, or a light application of the salt itself, will sometimes prove useful, particularly when the structure is accompanied with a chronic discharge. The injection should be sent in and returned, taking care to prevent its going into the bladder, by pressing with the fingers behind the stricture. This application has been successful also in chronic luecorrheas. *Scarifications* are now entirely out of date. No prudent man will make use of a means so injurious in its results, and so dangerous in the tube of the urethra.

*Progressive dilitation*, by means of ordinary bougies, is now almost the only means to which practitioners direct their attention. Cauterization, as described above, is sometimes added to this. — *Ibid.*

4. *New facts relative to the action of creosote, and to its theraputic value.*—Besides the now well known efficacy of this application to carious teeth, several cases of which are related by M. Fremanger, it has been found by this gentleman to be very useful in ulcerations of other bones. He cured with it a scrofulous caries of the osseous extremities of the first and second phalanges of the indicator finger of the right hand, with a fistulous wound traversing the articulation. This case had resisted iodine and mercurial preparations for six months; but in less than two months, a radical cure was obtained by the creosote. A seton of cotton was passed through the fistula, which was kept moistened with pure creosote for six days: this was then removed, and injections of strong creosote water employed, (15 drops to the ounce,) which was finally withdrawn for the following:

Cerate, ℥i.

Oil of sweet almonds, ℥i.

Creosote, gtt. xxx.

by which its action was for a long time felt.

M. Fremanger found that creosote has the property of opposing immediate union, which he supposes is occasioned by an inorganic pellicle constantly forming on the surface of the wound. — *Ibid.*

5. *Of the action of tannin upon the organic salifiable bases, and the applications which may be derived from it for medicolegal researches.* By Ossian Henry.—Among the properties by which alkaloids or vegetable alkalies, are distinguished, is that of their precipitation by an infusion of nutgalls, a property which is common to them all. Whether it is tannin, or gallic acid, which produces this effect, is a question which has not been entirely settled.

By means of this substance, M. Henry has been enabled to ascertain with great certainty and facility the quantity of quinine and cinchonine contained in the bark of the quinquinas. He has remarked that tannin, in precipitating the vegetable alkalies, produces with them very voluminious white, caseous compounds, easily collected, and generally insoluble in cold water; whereas, when potash, soda, chalk, or amonia is used, large quantities of them disappear, altered and dissolved.

The action of tannin upon the organic salifiable bases is precisely analogous to its action upon metalic oxides; that is, it combines with them. producing true salts. These saline compounds may be analyzed either by gelatine or humid parchment, or by double decomposition, with the aid of salts of tin, lead, antimony, peroxide of iron; or better still, by barytes, chalk or magnesia. The tannin is separated, and combines with the animal matter, or with the metallic oxides; and the alkaloid being set at liberty, either remains free, or unites with the acid of the decomposed metallic salts. In acting thus upon the aqueous solutions, diluted and acidulated with one or two drops of sulphuric acid, he found quinine, cinchonine morphine, codeine, narcotine strychine, brucine veratrine, delphine and emetine, with all their primitive characters. These are, doubtless, all bitannates, formed by the union of one atom of the base with two of tannic acid.

These salts are formed by adding an aqueous solution of pure tannin, or the extract of gall-nuts, or even any other liquid containing tannin, to a solution of a salt with a vegetable alkaline base, diluted, with slightly acidulated water.—*Ibid.*

6. *Notice of some experiments on the connexion between the Nervous System, and the Irritability of Muscles in Living Animals.* By Dr. J. Reid. With Observations by Dr. Alison.—Although physiologists are still divided in opinion as to the question whether nerves furnish a condition necessary to the irritation of muscles, (*i. e.* whether every stimulus which excites a muscle to contractions acts on it through the intervention of nervous filaments,) they have now very generally abandoned the once prevalent theory, that the irritability of muscles is derived from the brain or spinal cord, *i. e.* that muscles are continually receiving, through their nerves, from those larger masses of the nervous system, supplies of a certain influence or energy, which enables them to contract; and that some of the statements of Dr. Wilson Philip, in particular, are generally regarded as decisive against this theory.

Dr. Wilson Philip found by experiment, that the irritability of a muscle of which the nerves were entire, was exhausted by applying a stimulus directly to the muscular fibres, (sprinkling salt on them,) even more quickly than that of a muscle of which the nerves had been cut, and where all communication with the supposed source of nervous influence or energy had been cut off; and he states generally that a muscle of voluntary motion, if exhausted by stimulation, will recover its irritability by rest, although all its nerves have been divided.

But in opposition to this statement, and in support of the old theory of nervous influence continually flowing through certain of the nerves into the muscles, it has lately been stated by Mr. J. W. Earle, that when the nerves of the limb of a frog were cut, the skin stripped off, and the muscles irritated by sprinkling salt on their fibres, until they had lost their power of contraction, although they did not lose their power much more quickly than when the nerves were entire, yet *they did not regain their power*, although left undisturbed for five weeks; while the muscles of the limbs of another frog, similarly treated, but of which the nerves were left entire, completely recovered their irritability.

It occurred as a fundamental objection to the experiment of Mr. Earle, that in the case where the nerves had been divided, the muscles had become inflamed; being found at the end of the five weeks "softer in their texture than natural, a good deal injected with blood, and with some interstitial deposition of fluid in them;" while in the limb to which the salt had been applied, but of which the nerves were left entire, and where the irritability was recovered, "although the colour of the muscles was rather darker than natural, their texture remained unchanged, and there was no interstitial deposition of fluid in them."

In these circumstances it might evidently be supposed that it was the inflammation and disorganization of the muscles, not the section of the nerves, which prevented the recovery of the irritability in the case where the nerves had been cut; and it became important to have the experiment repeated, with care to avoid such injury of the limb of the animal as should cause inflammation to succeed the section of the nerves.

With this view Dr. Reid performed a number of experiments on frogs, in which the irritability of the muscles of both hind-legs were exhausted or greatly diminished by galvanism, after the nerves of one leg had been divided and the lower part of the limb rendered perfectly insensible and incapable of voluntary motion, (but without stripping off the skin,) while the nerves of the other had been left entire. The state of the muscles of both limbs was examined after some days. The results of these experiments were not uniform, but in several, where every attention to accuracy seemed to have been paid, the irritability of the muscles in the palsied limbs appeared to be *restored as perfectly as in the others;* contractions being excited in them, in several instances, by the galvanism from four or even two plates; whereas, they had formerly been irritated until they were no longer excitable by that from fourteen plates.

That the muscles which thus recovered their irritability had lost all nervous connexion with the brain or spinal cord, was proved, not only by their obvious insensibility, but by afterwards cutting off the heads of the animals and forcing a probe along the spinal canal, which excited forcible contractions in all parts excepting the palsied limbs.

Dr. Alison's paper contained the details of several of these experiments; and he stated in conclusion, that a *positive* result in such an inquiry must always outweigh a *negative* one, (particularly where a source of fallacy attending the latter can be pointed out,) these experiments appear fully to justify the assertion of Dr. Wilson Philp, that a muscle of voluntary motion may recover its irritability by rest, although all its nerves be divided; and that they afford, perhaps, more direct evidence than any others in support of the doctrine of Haller, now generally admitted in this country, that the property of irritability in muscles is independent of any influence or energy continually flowing from the nervous system, although, like every other endowment of living animals, it is subjected to the control of causes which act primarily on that part of the living frame.

Dr. Allen Thomson expressed a doubt whether these experiments warrented the conclusion drawn from them, not because he acquiesced in the theory to which they are opposed, nor because he called in question the accuracy of the results described to have been obtained, but because he knew that former experiments had failed in producing such diminution or exhaustion of the irritability of muscles as had been found by Dr. Reid; and conceived it possible that some of the numerous fallacies to which such experiments are liable, might not have been sufficiently guarded against.*

The accuracy of Dr. Reid's statement as to the great diminution or apparent exhaustion of the irritability of the muscles under the influence of the galvanism, and the subsequent recovery of the power, notwithstanding the devision of all their nerves, was satisfactorily established. It is to be remarked, however, that in these experiments, as usual in such cases, the limbs to which the galvanism was applied were kept moist by the same saline solution with which the galvanic trough was charged; and Dr. Thomson has observed, that when they are moistened with pure water, the diminution of the irritability under the excitement by galvanism is much less obvious. Hence he was led to suspect that the apparent loss of power in the muscles under that process might depend, not on the circumstance of repeated excitement, but on a degree, however slight, of injury to their texture by the action of the salt. This inquiry he proposes to prosecute farther; but in the mean time it is certain that by the usual process of galvanizing a living muscle moistened by a saline solution, a very great diminution of its irritability may be effected, which may subsequently be regained, notwithstanding the division of all its nerves; and as the fact of its recovery, not the cause of its dimination or exhaustion, is the point on which the inference drawn from these experiments rests, that inference may be held to be sufficiently justified.—*Fourth Report of British Association for the Advancement of Science.* —*American Journal of the Medical Sciences, November*, 1835.

7. *Notice of some Observations on the vital properties of Arteries leading to inflamed parts.* By Dr. Alison.—These observations were made with the able assistance of Mr. Dick, veterinary surgeon, on the arteries of the limbs of several horses, condemned on account of injury and inflammation there.

The immediate object of inquiry was, whether the totuous and strongly pulsating arteries leading to an inflamed part are really endowed with a greater vital power of contraction than sound arteries; and the method taken to ascertain this was to make a comparative examination of the condition of these arteries, and of the corresponding arteries in the opposite sound limbs, immediately on the animals being killed, (by blowing air into their veins;) and again after the lapse of 16 or 24 hours, when it is known that the tonic contraction, which takes place at the time of death, and is the indication of

*A Committee, of which Dr. Thomson was a member, was appointed for the repetition of the experiments, which has performed the duty assigned to it.

the only vital power which experiments authorize us to ascribe to arteries, has relaxed.

The animals were killed, and the observations made, at different periods varying from twelve hours to twenty days after the commencement of the inflammation, in the five cases of which an account was read. The extent of the inflammation was various. In all the cases, the artery leading to the inflamed part, when laid bare as high as the groin as soon as possible after the death of the animal, was larger in its whole length, *i. e.* had contracted less at the moment of death, than that of the sound limb. In two of the cases, where the inflammation was of long standing, and the coats of the artery appeared to have been effected by it, this vessel at the second examination appeared smaller than the artery of the sound limb, having not only contracted less at the moment of death, but dilated less after death, than the artery in the natural state. In the other cases the artery of the inflamed limb remained larger than the other at the second examination; and it was farther obvious that its elasticity was impaired, for when slit open and smoothed out, it had less power than the sound artery of recovering the cylindrical form.

In all the cases, the artery of the inflamed limb retained after death a considerable quantity of blood, while the other was almost empty; and that this was not owing to inflammatory effusion, preventing the artery of the effected limb from emptying itself at the time of death, was proved, in two of the cases, by cutting across the vessel, immediately on the death of the animal, a little above the inflamed part, whereby it had full opportunity to rid itself of its blood, if it had retained the power to do so.

One of these observations was made in the presence of Dr. Yellory, Dr. Clark, Dr. Fletcher, Mr. Broughton, Mr. Clift, and Mr. Bracy Clark: and it may be added here, that in a subsequent experiment, in which Dr. Alison and Mr. Dick were assisted by Dr. Fletcher. they obtained farther proof of the loss of elasticity in the artery of an inflamed limb, by finding that after it hed been distended by a given weight of mercury, (in the way practised by Poiseville,) it had less power than the corresponding sound artery, to contract on itself and expel its contents when the distending force was withdrawn. But this last experiment was made too long after the death of the animal to justify an inference as to the strictly vital power of the vessel.

Dr. Alison stated, that it seems now generally admitted by microscopical observers, that during by far the greatest part, and during the highest intensity of inflammation, nothing but dilitation or relaxation of the *small* vessels of the inflamed part can be perceived. If the present observations shall be confirmed by others, they will show more distinctly than any statements hitherto on record, that the same holds true of the *larger* vessels supplying an inflamed part. Now, there are two changes in the movement of the blood through the vessels of an inflamed part which seem well ascertained by many observations, viz: retarded movement or absolute stagnation, (*stase du sang*,) in many of the small vessels most affected, even during the height of the inflammation; and accelerated movement in the neighboring vessels, with greatly increased transmission; in a given time, through the whole veins of the part. This last change may, perhaps, be reasonably ascribed to the relaxation of the vessels giving increased effect to the impulse from the heart; but it seems impossible to ascribe likewise to that relaxation of vessels, the former which is just the opposite change in the movement of the blood; and yet no modification of the action of any of the vessels, except simple relaxation, can be detected.

The fair inference from these facts therefore seems to be, that the phænomena of inflammation are truly inexplicable by any changes which occur, during that state, in the contractile power of the vessels containing the blood; and that, instead of seeking for an explanation of these phænomena in the state of *contractions* of any of the solids, we ought rather to look for it in the state of the *attractions* subsisting during the living state among the particles of the blood, and between them and the surrounding solids. And this inference the author thinks might be supported by reference both to other facts in the history of inflammation, and also to many other phænomena of the living body, both in health and disease.—*Fourth Rep. of British Association for the Advancement of Science.—Ibid.*

8. *Report of Progress made in an Experimental Inquiry regarding the Sensibilities of the Cerebral Nerves, recommended at the last Meeting of the Association.* By Marshall Hall and Mr. Broughton.—Some disagreement appears to exist amongst the results of the investigations regarding the sensibilities of the cerebral nerves, which demands farther experimental inquiry. A series of experiments has therefore been instituted at the request of the Committee of the Medical Section, and the establishment of Messrs. Field in Oxford street, London, was selected for the purpose of carrying the inquiry into effect; the horse and the ass, from their large size, being considered as the most favorable subjects for the free exposure of the nerves.

The properties of some of the cerebral nerves being admitted upon other grounds than experimental proof, this investigation was exclusively directed to the facial branches of the fifth pair of nerves, the hard portion of the seventh, the vagus, the spinal accessory, the glosso-pharyngeal, the lingual, and the sympathetic nerves. Upon the properties of the first, second, third, fourth, sixth, and the soft portion of the seventh pair of nerves no doubt or discrepancy exists.

It has long been known that the properties of the cerebral nerves are various. Thus, one nerve governs the function of motion; another that of some specific sensation, as of light or sound; and these properties are held independently of each other. To understand clearly the properties of nerves, it is also necessary to apply the test of experiment to their *roots;* for branches from two or more roots unite to form one nerve apparently, which may then assume two distinct properties, that is, the peculiar property of each root. This is exemplified in the origin and distribution of the nerves of the face.

The apparent discrepancies in the results of experiments probably depend much upon the indefinite manner in which certain physiological terms have been employed. Thus, *sensation* has been coupled *with* consciousness in some instances, and in others it has been supposed to exist *without* consciousness. In the present report the term *sensation* implies *consciousness.* It is considered as identical with *feeling,* and when violently excited it becomes *pain.* And this is manifested by *general and instantaneous* efforts or struggles. *These are, therefore, the signs of sensibility.*

Three modes of judgment have appeared as necessary to be kept in view in the present inquiry in reference to the above definition:

1. It was observed that when a nerve of unequivocal sensibility was pricked or pinched, an *immediate and general* struggle followed. The facial branches of the fifth nerve are examples.

2. That when a nerve as unequivocally devoted to motion is pinched, there is an *immediate contraction of the muscles which that nerve supplies, and of no other muscles.*

3. That on pinching the par vagum, neither of the phænomena above noticed occurs; but by continuing the compression for a few moments, an *act of respiration and of deglutition follows, with a tendency to struggle and cough.*

Of these three phænomena the first only is considered as indicating the property of sensation, or the power in the nerve subjected to experiment to transmit sensible impressions.

The movements in the third instance appear to arise from secondary causes, *the mechanical irritation of the nerve not being attended with immediate consciousness.*

1. *Experiments upon the Facial Nerves.*—These nerves govern the actions of the face, and preside over the sensibilities of its different organs and surfaces. The first function is performed by the facial portion of the seventh nerve and a portion of the fifth. The second function is performed by the large portion of the fifth pair of nerves. Thus the fifth nerve possesses two distinct properties of transmission, one voluntary, the other sentient, in consequence of its having two distinct roots. One of these roots, the largest, has a ganglion attached to it, and is exclusively a sentient nerve. The smaller root has no ganglion, is insensible, and governs the motions of those muscles which it supplies. The first fact is easily gained by experiment, but the second is admitted upon other grounds, for the smaller root cannot be experimented upon in the living animal. It is to be observed that the larger root of the fifth nerve is divided into three branches, spread and ramified over the face, and frequently connected in its ramifications with branches of the seventh nerve; so that unless the experimental tests be applied to distinct branches, no certain response can be obtained as to their several properties.

Pricking or pinching the trifacial nerve was attended with instantaneous indications of consciousness; when its branches were divided, all sensibility ceased in the parts which they supply. The *lower* divided ends made no response when bruised, that the upper indicated sensation. The motions of the face, however, still remained unimpaired, until the seventh nerve was divided as near its origin as possible, when the organs which it supplies became permanently motionless. When this nerve was slightly pinched in its entire state, those muscles exclusively which it supplies were seen to be convulsed, without any *general* effort; when the compression was increased, and continued for a few moments, signs of uneasy respiration occurred. Pricking this nerve with a needle and cutting through it produced no struggle whatever, as is the case with the trifacial nerve. When the lower end of the nerve, after division was irritated, no movement followed; but on compressing the upper end, the same signs were exhibited as when the nerve was irritated in its entire state.

2. *Experiments upon the Nervus Vagus.*—In the year 1820, Mr. Broughton experimented upon this nerve: the results were published in the *Quarterly Journal of Science of the Royal Institution.* It was found to be insensible when slightly pinched, pricked, or divided. The present experimental investigation confirmed this remark. It was also on the former, as well as upon the recent occasion, clearly shown that, when a forcible compression was continued a few moments upon the nervus vagus, a respiratory effort followed, and an act of deglution, with a cough and a struggle. In the recent investigation it was observable that when the nervus vagus was divided, mechanical irritation applied to the upper end of the divided nerve produced the same signs as when the nerve was entire. Every repeated compression of this nerve, (as was also the case with the seventh,) produced corresponding respiratory struggles; whilst a uniform, uninterrupted compression caused no repetition of the phænomena. An additional argument in support of the opinion that these effects are independent of any sensible property in the nerve itself is furnished by the fact that Dr. Marshall Hall has found precisely similar effects to occur in the turtle after its decapitation, on pricking the laterai spinal nerves, whether of the sentient or motory class.

3. *Experiments upon the Spinal Accessary Nerve.*—This nerve having been pricked without any response, was then slightly pinched and scraped; when the sterno-maxillaris muscle, the levator humeri, and other muscles of the neck exclusively were seen to contract at each application of this mechanical irritation. But when the forceps was applied firmly, and continued a few moments, similar effects were produced as with the vagus and the seventh. The branches of this nerve appeared to be equally destitute of sensibility with the root. The compression of the upper end, after dividing this nerve below its bifurcation, was followed by no effects, unless the pressure was made opposite the giving off of the anterior branch, when the same phænomena occurred as were exhibited in the entire nerve.

4. *Experiments upon the Glosso-Pharyngeal Nerve.*—When this nerve was pricked, scraped, or divided, no response was observed. The muscles of the root of the tongue were most probably set in motion by the compression of this nerve at intervals; but no opportunity occurred of bringing this part of the tongue into view. Neither in its entire nor divided state did any struggle arise from the continued compression of this nerve, which is therefore regarded as one simply of muscular motion.

5. *Experiments upon the Ninth Nerve.*—The sensible surface of the tongue is supplied by the ganglionic portion of the fifth nerve, whilst the muscles of its fore part are furnished with branches from the ninth nerve. No sign of sensation was evinced by mechanically irritating the trunk of this nerve, and its division was unattended with any sign of feeling or pain. But upon pinching it slightly at intervals, those muscles which it supplies, on the same side of the tongue, were convulsed. If the nerve was forcibly compressed, a slight gulp followed. When the nerve was divided, pinching the upper end of it was not followed by any muscular contractions.

6. *Experiments upon the Sympathetic Nerve.*—No experiments upon this nerve have hitherto exhibited any signs of sensation or muscular motion of any kind whatever. Its division is never followed by any visible effect.

*Remarks.*—By these observations some researches of other experimenters stand confirmed, whilst others are contradicted; the necessary consequence of discrepancies, often arising from the different modes of applying certain terms. Although the mysterious properties and actions of the nerves may never be completely unravelled, yet much has been effected by the successive and combined efforts of physiologists of different ages and contries.

The present investigation leads to the assumption, that one only of those nerves which derive their roots from the brain itself is, according to the definition laid down, a nerve of sensation. This is the larger and ganglionic division of the fifth nerve, whereby animals are enabled to examine by touch and to feel.

With regard to the other nerves subjected to experiment in this inquiry, none of them appear to possess in themselves any power to excite consciousness or feeling directly. Some of them are simply nerves of motion, and they transmit no other impressions but such as excite local musclar motion, limited to the muscles which they supply. Others, again, seem to possess a property of a different description from either of the two former kinds. One of these, the eighth for example, appears to be so intimately connected with the respiratory function as to be capable of influencing it in a most remarkable degree, without exhibiting any sign of sensation in itself, or of simple and direct muscular contraction.

It is a most remarkable fact, that when a nerve which influences respiration is divided, and the upper division is bruised or compressed for a few seconds, the same effects occur as when the irritation is applied to the entire nerve. This phænomena affords matter of curious and interesting speculation with regard to the relations which subsist between the nervous and the respiratory functions.

The farther pursuit of this inquiry may lead to some farther development of facts hitherto exposed in some instances to doubt and controversial discussion.

Dr. Hall was necessarily absent at one of the experiments, that on the ninth nerve; but he feels perfectly satisfied with the joint testimony of Mr. Field and Mr. Broughton.—*Fourth Report of British Association for the Advancement of Science.—Ibid.*

9. *Inquiries into the Varieties of Mechanism by which the Blood may be accelerated or retarded in the Arterial and Venous Systems of Mammalia.* By Dr. T. J. Aitkin—The attention of the Section was particularly directed to four modifications of arterial distribution, as indicated, (1.) by the angle at which a branch comes off from its trunk; (2.) the direction of the vessel; (3.) the subdivision; and (4.) the formation of plexus.

In illustration of the first, or angle of origin, Dr. Aitkin exhibited a preparation of the aorta of the tiger, in which the superior intercostals arose at an acute, the middle at a right, and the lower at an obtuse angle; from which he inferred that the force and velocity of the blood are rendered equal through the whole series. In speaking of the direction of the vessels, he adverted to the tortuous entrance of the internal carotid and vertebral arteries into the skull in the human subject, and showed that it is still more remarkable in the horse, which in feeding requires to have the head for a considerable time in the dependent posture. But the best examples of the tortuous or serpentine course are to be seen in the spermatic arteries of the *Mammalia*. This mechanism, the author contends, adapts the circulation to the various positions in which organs may be placed, and to their states of action and repose. In speaking of the third modification, or the subdivision into numerous long branches, he particularly alluded to the observations of Sir A. Carlisle with respect to the arteries of the sloth, and showed that a similar ramification is found in the hedge-hog, both in the arteries of the panniculus carnosus and of the mesentery. Of the last modification, the plexus, he showed examples in the rete mirabile of Galen in the internal carotid, and of Hovius in the opthalmic artery, of the *Ruminantia*. He inferred that this structure prevents valvular turgescence, which would otherwise occur during the long period these animals keep their head in the dependent position while browsing. He also showed, that a rete mirabile exists in the opthalmic artery of the seal and goose, and considered it probable that in them it is conducive to the alternate adaptation of the eye to vision in air and water. He described the remarkable plexiform arrangement which exists in the mesenteric arteries and veins of the hog; and instituted a comparison between those vessels in carnivorous and herbivorous *Mammalia*, concluding that these modifications are in conformity with the transmission of blood through the liver, the rapidity of the peristaltic motion, and the power of nutrition.—*Fourth Report of British Association for the Advancement of Science.—Ibid.*

10. *Pathology of Apoplexy.* By William Stokes, M. D.—The term apoplexy, as I suppose you all know, is derived from a Greek word, signifying a stroke or blow. It is a term which, in the present state of medicine, has been very frequently abused, or at least employed in very different senses, and hence the many erroneous opinions respecting it. The true meaning of the term expresses an alteration of the phenomena of the life of relation, that is, of the functions of the cerebro-spinal system. In taking a view of the nature of this alteration, we find that the attack generally comes on in a sudden manner, and that the functions of the brain are partially, or completely suspended. You are aware that the manifest phenomena of the life of relation are those which belong to *sensation*, *muscular motion*, and *the intellect*, and that the system of the life of relation is composed of the brain, spinal cord, and nerves. Now suppose, for example, that a man gets an attack of apoplexy, we find him paralytic—here is a lesion of the muscular function. We find him insensible to external stimulants, he feels no pain—here is a lesion of sensation. We may find his sight, hearing, taste, smell, and touch are injured. He lies in a state of insensibility, and is unconscious of every thing passing around him—here we have an example of interruption in the performance of the intellectual functions. All these phenomena exhibit the various lesions superinduced by an attack of apoplexy in the functions of those organs which subserve to the life of relation.

I have said that the term apoplexy is frequently abused in modern medicine. From the circumstance of most cases being accompanied by an effusion of blood on the surface, or into the substance of the brain, the term has been also applied to sanguineous effusions into other organs, and we hear every day of pulmonary and hepatic apoplexy; terms implying the extravasation of blood into the substance of the lung or liver. The analogy, however, in such cases will on examination he found to be coarse, and the application of the term loose and improper. Apoplexy, as a cerebral disease, may occur with or without effusion; in either case the disease, *quoad* the lesion of function, is the same; but to give the name of apoplexy to hæmorrhage into the lungs or liver is improper. The term apoplexy should be used only with reference to the brain, and applied to a particular train of lesions in the functions of the life of relation occuring *with or without an effusion of blood, or even congestion.* When we have effusions of blood into other viscera, we may have them unaccompanied by any apparent lesion in the functions of the organ affected, (a circumstance rarely met with in the case of the brain,) and it would be much better to give some other name to those hæmorrhages into the substance of the liver and lungs, than to designate them by one drawn from a loose and imperfect analogy.

The suspension of the phenomena of the life of relation, complete or partail, which constitutes apoplexy, may be connected with any of the following pathological conditions. First, great congestion of the brain, in which the vascular system of that organ is overloaded, but without extravasation of blood or serum; this is termed *the congestive apoplexy.* In the next place, we may have this congested state of the vessels of the brain with an extravasation of blood on its surface. To the latter form the *meningeal apoplexy* has been applied. Thirdly, with an effusion of blood into the substance of the brain, which is the most common case; and, lastly, we may have complete apoplexy, without morbid appearance, or, if there be such, *quite insufficient to account for the phenomena.* A man will fall down suddenly, he will lie in a state of insensibility, with stertorous breathing, coma, and paralysis, he will die with all the symptoms of the worst form of apoplexy, and yet on dissection the brain may be found *to all appearance healthy.* This is what has been termed by the older authors the nervous or convulsive apoplexy, of the real nature of which we are still as ignorant as we are of the nature of tetanus, hydrophobia, and other nervous diseases unaccompanied by perceptible organic change.

This is the *simple apoplexy* of Dr. Abercrombie, of which he gives several most important cases, and refers to others related by the older authors. You will at once admit that it is not more extraordinary that apoplexy should exist without perceptible organic change than mania, tetanus, hydrophobia, and other affections. Of the fact there is no doubt. Such cases indeed are rare, which in one sense may be looked on as a fortunate circumstance. But in the progress of other diseases, this nervous coma or apoplexy is by no means uncommon. Thus there is no symptom more common than coma in typhus, and yet if you examine the head after death, you generally either find no lesion at all, or such as will not be sufficient to account for the symptoms. The coma, which occurs in cases of painters'

colic too, appears to be closely connected with this nervous apoplexy. You will recollect an interesting clinical experiment I made in the case of a patient with painters' colic who had profound coma. In this case I thought it probable that the condition of the brain bore no resemblance to sanguineous apoplexy, because the symptoms of painters' colic are seldom or never accompanied by hyperæmia of the nervous or other systems. Under this impression, I prescribed a full opiate, and this not only did not increase the coma, but, on the contrary, produced the very best effect, for the patient was amazingly improved the next morning. I do not so much mean to say, that opium is useful in nervous coma, as that in this instance, at least, the coma was not of the congestive kind. It is not unlikely, too, that the coma of jaundice is of the same description and unconnected with any decided hyperæmia of the brain. I am aware that in jaundice the coma is supposed by some to depend upon a bilious condition of the blood circulating in the brain, but there are so many cases of persons who have laboured under jaundice for years without having coma, that we must seek for some other explanation. Now, so far as we know of the encephalon in persons who have died of jaundice, it appears that little or no congestion exists, and hence it seems probable that the coma of jaundice is similar to that of nervous apoplexy.

I shall now proceed to the consideration of those forms of apoplexy which are connected with changes more or less apparent in the circulation of the head, and with which we are consequently better acquainted. I have told you that simple congestion of the brain may be accompanied by symptoms of apoplexy, or that we may have the disease presenting in addition to this an effusion of blood into the substance, or on the surface of the brain. The simplest idea you can get of the condition of the brain in the congestive form, is to consider what its state is in persons who have been hanged. These persons have the vessels of the brain loaded with blood from the violent interruption of the venous circulation. Now, this increase in the quantity of blood circulating in the brain may arise from two causes, one depending on the interruption of the venous circulation, the other produced by an increased action of the arterial system. Hence in certain cases of disease of the heart, where the blood is sent with great force to the head, there is a strong predisposition to apoplectic attacks. The kind of disease of the heart, however, which has been found most liable to produce this, is not, as you would suppose, Corvisart's active aneurism, but simple hypertrophy of the heart, where the cavity of the left ventricle continuing the same, its walls are increased in thickness and strength, so that on the natural quantity of fluid an increased impulse is exercised. Such at least is the result of Andral's researches, and there is every reason to place confidence in the accuracy of his conclusion.

About this congestive apoplexy there appears to have been a good deal of misapprehension. You have all heard of *the serous apoplexy*. In this form it has been supposed that the cause of the compression of the brain and all the other symptoms is an effusion of serum into the cavity of the pleura, which will produce compression of the lungs and dyspnœa. The idea which has been generally entertained is, that the effusion of serum is the cause of *all* the symptoms, and, in consequence, the same active treatment has not been adopted as in the other forms of apoplexy. This opinion will be best refuted by the investigations of Dr. Abercrombie, and I cannot do better than read for you the opinions of this eminent writer on the subject, as given in his celebrated and admirable work, which I have no hesitation in saying constitutes one of the brightest ornaments of British medicine.

"The distinction, which has been proposed betwixt sanguineous and serous apoplexy, is not supported by observation. The former is said to be distinguished by flushing of the countenance and strong pulse, and by occurring to persons in the vigor of life; the latter by paleness of the countenance and weakness of the pulse, and by affecting the aged and infirm; and much importance has been attached to this distinction, upon the ground that the practice which is proper and necessary in the one case would be improper or injurious in the other. I submit that this distinction is not founded upon observation, for, in point of fact, it will be found that many of the cases, which terminate by serous effusion, exhibit in their early stages all the symptoms which have been assigned to the sanguineous apoplexy, while many of the cases, which are accompanied by paleness of the countenance and feebleness of the pulse, will be found to be purely sanguineous; and one modification of the disease in particular will be described, in which these symptoms are very strikingly exhibited, while the disease is found to be sanguineous apoplexy in its most hopeless form.

"Portal has described a series of cases which afford the same result; of three, which presented all the symptoms of serous apoplexy, one was saved by repeated bleeding, and in the other two, which were fatal, there was found extensive extravasation of blood. Case XCVI., lately described. forms a remarkable addition to these observations. If any case could be confidently considered as serous apoplexy, this was such. Dropsical effusion had existed in the body for months, and in defiance of every remedy it had been progressively gaining ground. There were symptoms indicating its existence, both in the thorax and in the abdomen; the patient then became comatose with pale countenance, and died; but though dropsy was found in the other cavities, none could be detected in the brain.

"In other parts of the body serous effusion is very seldom a primary disease; it arises as a result either of inflammatory action, or of impeded circulation, and takes place slowly, not accumulating at once in such quantity as to induce urgent symptoms. It is therefore in the highest degree improbable, that it should occur in the brain as a primary disease, and accumulate with such rapidity as to produce the symptoms of an apoplectic attack.

"The quantity of fluid effused bears no proportion to the degree of the apoplectic symptoms. We find it in small quantity, though the apoplectic symptoms had been strongly marked and long continued; we find it in large quantity when the symptoms have been slight; and finally, we find most extensive effusion in the brain where there have been no apoplectic symptoms at all. The direct inference from these facts is, that, in the cases of apoplexy with effusion, the presence of the fluid cannot be considered as the cause of the apoplectic symptoms."

The same error has been committed with respect to hydrothorax, a disease almost never primary, but the result of either pleuritic inflammation, obstruction of the heart or lungs, or some analogous cause. The cause of the symptoms is not the mere effusion of fluid, but some preexisting disease which has given rise to a serous effusion. In Dr. Abercrombie's work you will find the remarkable fact stated, that there may be a copious effusion of serum in the head without producing apoplectic symptoms. The following case, mentioned by Dr. Abercrombie, furnishes a remarkable illustration:—A patient, who had labored under hypochondriasis for upwards of thirty years, began to decline rapidly in health. He was extremely feeble, his bowels costive, his sleep disturbed, and his appetite gone. This state continued for some time, and he began to sink, but he never complained of head-ache, giddiness, convulsions, or paralysis, and his mental powers remained unimpaired until a very short time before death. Yet, on opening the head, there was an exceedingly copious effusion of serum found under the arachnoid, and in some places this was so great as to give the arachnoid the appearance of small bladders filled with water. The ventricles were distended with fluid. Dr. Abercrombie gives another case, where the quantity amounted to eight ounces, and notices a case, mentioned by Dr. Marshall, of a maniac who died of mortification of the feet; a few hours before death he became perfectly rational, yet effusion was found both on the surface of the brain and in the ventricles, amounting to more than a pound.

All these facts go to prove, that what has been termed serous apoplexy is only an apoplectic attack depending on congestion of the brain, that in some cases we may have this congestion accompanied by serous effusion, in others not; that the effusion is secondary and by no means of constant occurrence, and that altering our practice and pursuing a less active plan of treatment in such cases would be improper. The same treatment should be adopted in the serous as in the congestive form of the disease, for where the nature of the affection is the same, the same curative means should be employed. Why it is that effusion takes place in one case and not in another we cannot tell: such changes are connected with laws of organization, of which we are at present ignorant. We know as little why this should occur as why inflammation of the liver in one case is followed by enlargement, in another by the secretion of pus, in a third by cancer, or in a fourth by hydatids.

We now come to the consideration of apoplexy with extravasation of blood. This is the form of the disease to which the term apoplexy has been restricted by one of the last writers on the subject, M. Rochoux. In this affection the extravasation of blood, which constitutes the principal pathological feature of the disease, is found to exhibit a remarkable variety as to its seat and extent. In some cases the blood is effused on the surface of the brain, in others into its substance, and in a few cases into the ventricles. De Haen gives some cases of apoplexy produced by rupture of the choroid plexus, but in the great majority of cases, where blood is found in the ventricles, the extravasation has taken place in one hemisphere, and, tearing through the substance of the brain, has made its way into their cavities. Of the three varieties of apoplectic effusions, the vantricular is the rarest; the next to this is the meningeal, or that in which the blood is poured out on the surface of the brain. and the most common is where it is effused into the substance. It has been also found that certain parts of the brain are much more liable to sanguineous effusions than others; of the reason of this, as of many other phenomena connected with the circulation of the brain, we are still in ignorance. The following table, which you should bear in mind, exhibits remarkable preponderance in the liability to sanguineous effusions of certain parts of the brain. It has been taken from the Precis d'Anatomie Pathologique of Andral. The following is a summary of the results of 386 cases of apoplexy.

In 202 cases, the effusion took place into the substance of the hemispheres of the brain, in that part which on a level with the corpora striata and optic thalami. The portions of the brain next most liable to effusions are the corpora striata; and here we have 61 cases. Next to this are the optic thalami, in which we have 35 cases. In that portion of the hemispheres above the centrum ovale, 27 cases. Lateral lobes of the cerebellum, a proportion of 16 cases. In those portions of the brain anterior to the corpus striatum, 10 cases. In the mesocephalon, 9. Spinal cord, 8. Posterior lobes of the brain, 7. Middle lobe of the cerebellum, 5. Peduncles of the brain, 3. Olivary bodies, peduncles of the cerebellum, and pituitary gland, 1 in each, making 3.—Total 386. Out of these, we find 325 cases occurring in the hemispheres of the brain, corpus striatum, and optic thalamus.

In the number and size of these effusions we find the greatest varieties. In some cases an enormous effusion takes place, and many ounces are extravasated into the substance of the brain; in others, the quantity is trifling, being sometimes as small as a pea, or even less. It has been observed that in cases where numerous extravasations were discovered, they were generally found to be in different states, as if they had occurred at intervals, and not simultaneously. This leads us to the knowledge of one of the most important facts in pathology, that in many cases of apoplexy, after a clot has been formed, nature commences at an early period a process of cure. The change, which takes place in cases where a patient recovers, seems to be the following:—It becomes at first somewhat gelatinous, it is next observed to be more consistent, and it loses its red color, and takes on a whitish or yellow appearance. The clot is gradually removed, and along with the absorption of the clot there is a process of isolation going on. A fine membranous cyst, furnished with vessels, is formed round the clot. In some cases the clot is replaced by a quantity of serous or gelatinous fluid; but in the majority of instances this does not occur, and the cyst has been found empty. This is a fact which has been established by numerous observations.

There is the greatest possible difference as to the period at which the absorption of the clot is completed; but we may safely assert, from the number of cases in which, after paralysis, a recovery takes place, that this process is of very common occurrence. In several cases, where apoplexy followed by paralysis has happened several times during the lifetime of the patient, a number of those cysts, corresponding with the number of attacks, and presenting various appearances according to the date of their formation, have been found. It appears then that the cure of apoplexy depends solely on the absorption of the clot; and that, as long as this remains unabsorbed, the patient is in danger. In some cases absorption does not take place at all, the clot becomes organized; and in this way it is supposed that some of the tumours found in the brain are formed. There are several circumstances which favor the absorption of the clot, but nothing so powerfully as a healthy condition of the whole cerebral circulation. This leads us to the consideration of the importance of paying attention to the head long after an attack of apoplexy. It inculcates the necessity of avoiding every thing calculated to add to the existing congestion, and shows that, in the paralytic or after-stage of an apoplectic attack, we should not neglect to deplete the head from time to time. The great point is to keep the head perfectly free from irritation; for it has been found, that, where a cure appeared to be going on, any new irritation applied to the brain has had the effect of arresting the absorption of the clot, and marring the process of cure.—*London Medical and Surgical Journal, July 26th*, 1834.—*Ibid.*

11. *Circumstances which predispose to Apoplexy.* By William Stokes, M. D.—Apoplexy with sanguineous effusion may occur at any period of life: it is not uncommon even in persons of tender years. Billiard details an instance of this in a child soon after birth. There are also several cases mentioned as occurring in children during the first three or four years. Andral gives the case of a boy of nine years of age, who died of apoplexy, with a vast effusion of blood. One of the most remarkable cases of this kind I ever witnessed, occurred in a child who had just been weaned. This child had been laboring for some time under symptoms resembling hydrocephalus, and then suddenly got an attack of convulsions, followed by coma and paralysis of one side. From a careful study of the symptoms, I ventured to make the diagnosis of apoplectic effusion, and on examining the brain after death, there were nearly three ounces of blood found effused in the base of the brain. But though apoplexy may occur at any poriod of life, there is a greater liability to it at a particular period. Rochoux has shown that the age at which there is this greatest liability, is towards sixty, and diminishes towards seventy. The number of cases which occur between sixty and seventy are very great, when compared with those between seventy and eighty; and after eighty he considers the liability to be still farther diminished. It seems strange, that persons after seventy should not be so liable to attacks of apoplexy as before that period, but such is the fact. It has been thought that this may be explained by the anemic state of the brain in old persons; it is said, that at such an advanced age general emaciation takes place, and the quantity of blood is greatly diminished. This explanation, however, is doubtful, because it is at present well ascertained, that persons of ordinary development, who are neither fat nor thin, and also persons of spare and delicate habit, are as much, and even more, liable to apoplexy than the fat and plethoric. It has been ascertained by careful investigations, that a high degree of plethora does not necessarily predispose to the disease, and that it is oftener met with in persons not of a plethoric habit than in those who are. These considerations throw some doubt on the opinion that an exemption from apoplectic attacks is connected with an anemic condition of the system. It generally happens, however, that at this advanced period of life, from the general debility of the system, and the incapacity for active exertion, a man ceases to employ his thoughts about business, and there is little exercise for the intellectual functions. We now have finished the task; the brain reposes from the turmoil of active and incessent thought; there is a comparative absence of mental exertion, and this may in some degree account for the rarity of apoplexy after the age of seventy.

With respect to the different temperaments as bearing on this point, Rochoux shows that in Paris, at least, there was a nearly equal frequency of the disease in individuals of the sanguine, sanguineo-bilious, and sanguineo-lymphatic constitutions. The bilious temperaments, however, are much less liable. Such is the result of the observations in Paris; but it must be recollected, as Rochoux observes, that in that city the bilious temperament is the rarest. With respect to the sanguine or plethoric, it has been found that this temperament does not predispose to apoplexy so much as has been generally supposed. The disease has been observed to be most common in persons of ordinary development, next to those in persons of thin, spare habit, *and last of all, in the plethoric and fat.* Rochoux's researches lead him to conclude that the number of persons of ordinary development, attacked by apoplexy, is three times that of the plethoric, and that that of the spare habit is little more than twice as great as that of the fat and plethoric. If these researches are correct, they afford great consolation to stout gentlemen.

The conclusion, which has been come to, with respect to temperaments as bearing on the liability to apoplexy, appears to be true; namely, that there is no sign appreciable by the senses which will unequivocally point out a predisposition to apoplexy. This is of great importance in a practical point of view. You may expect the disease in the fair or dark haired, the thin or fat, alike. The frequent occurrence of this disease in persons who were never suspected to have any predisposition to it is another proof in favor of this opinion.—*Ibid., August* 2*d*, 1834.—*Ibid.*

12. *Diagnosis of Apoplexy.* By William Stokes, M. D.—With respect to the mere medical diagnosis of apoplectic effusion, it would be well if, in making it, you would always bear in mind the anatomical characters of the disease. Extravasation of blood into the substance of the brain generally takes place by a tearing or separating of the cerebral tissue. A quantity of blood is rapidly effused, the substance of the brain torn, and a cavity formed. There can be no doubt that the tissue of the brain is torn, for we can see the loose shreds hanging on each side of the cavity, and mixed up with the clot. Now, what are the principles which should guide us in making our diagnosis? They are exactly the same as those in other diseases *connected with a sudden solution of continuity in the substance of internal organs.* We have, with or without any preceding symptoms of a different kind, the *sudden* supervention of new and remarkable phenomena. The phenomena which are the result of disease proceeding in its ordinary course are gradual and progressive; but occurrences of this kind are almost always characterized by sudden and well-defined symptoms. Thus, we make the diagnosis of the rupture of an aneurism of the aorta from the sudden vomiting or expectoration of blood, followed by the death of the patient. Here, you perceive, the diagnosis is founded on the *sudden* supervention of new symptoms. In the same way we may make the diagnosis of pneumothorax with a fistulous opening communicating with the bronchial tubes, and calculate from the sudden occurrence of pain in the side and the other signs of pneumothorax, that there has been a solution of continuity in the pleura. Again; if a person, laboring under hepatic abscess, is seized with a fit of coughing, and suddenly expectorates a quantity of pus, and that this is found to be accompanied by a subsidence of the tumour in the region af the liver, we make the diagnosis of perforation of the diaphragm and pleura, and the escape of the contents of the abscess into the substance of the lung. Or he may, under the same circumstances, be seized with sudden and rapid peritonitis, and here we make the diagnosis of an effusion into the peritoneum. It is on precisely the same principles that Louis has established the diagnosis of perforation of the small intestines in cases of gastro-enteritis. The patient is lying in bed, perhaps apparently improving; he is not exposed to any exciting cause, and every care may have been taken of him. *On a sudden* he exhibits symptoms of intense peritonitis, and rapidly dies. Any one conversant with such cases can easily make a correct diagnosis. On the same principles we found the diagnosis of apoplectic effusion. Almost all the instances of disease which I have given occur with a *sudden* violent invasion; and the same thing may be said of apoplexy with extravasation. It is true, that there are some cases which do not exhibit this character, but the general rule is suddenness of attack.

We may divide apoplectic attacks accompanied by extravasation into three great classes, and, if you look to the great majority of cases of this disease, you will find that, although they appear to pass by insensible degrees into one another, still, when taken and examined singly, there will be found a difference between them. This classification is that of Rostan, and I have known his principles verified in many instances. In the first class of cases, which are the worst and generally prove fatal, the extravasation is enormous. A person, apparently in perfect health, will fall down in a fit of apoplexy, remain for a short time insensible and paralytic, and then die. In such a case as this, the ordinary pathological character is an enormous effusion of blood, or excessive congestion. In a case of the second class, we have an apoplectic seizure with coma, which disappears after some time, and the patient recovers his intelligence, *but with paralysis of one side.* The pathological character of this form is, that the effusion is more limited, and exists only on one side of the brain. Neither is the congestion so severe, and the patient recovers from the coma. In the third form, we have an attack of apoplexy of a milder description; there is scarcely any coma or loss of intelligence, and the paralysis is slight, generally affecting the muscles of one side of the face, or of one of the extremities. Let us repeat these varieties. In the first, which constitutes the *apoplexie foudroÿante* of the French, there is an enormous extravasation of blood in both sides of the brain; or, if it be only on one side, the amount of the effusion is frequently such as to burst through the walls of the ventricles and get into their cavities, and in this way we may have an effusion of one side getting into the other hemisphere, or exercising such pressure on it as may give rise to *general* symptoms.

Such a case as this is, I believe, generally fatal; its progress, too, is very rapid, several persons under such circumstances having died in the space of an hour, or less. In the second form, there is coma and loss of intelligence, and the patient recovers *with paralysis of one side.* Here the extravasation is never so great as in the foregoing case; the effused blood is confined to one side, and does not get into the ventricles. In the third form, the effusion is very much circumscribed, the signs of general congestion or extravasation are slight, the quantity of blood poured out is not, perhaps, larger than a nut, it is followed by partial paralysis, and there is little or no coma or loss of intelligence.

Let us take a brief review of the symptoms which attend each of these forms. In a case of the first description, we find a person, hitherto in the enjoyment of health, suddenly attacked with symptoms of intense apoplexy. You will recollect that in my last lecture I told you that apoplexy consisted in various lesions of the phenomena of the life of relation. In the most violent form of apoplexy, many authors are of opinion that there is a total paralysis in the functions of animal life. The patient falls down and remains in a state of complete insensibility, the eye no longer obeys the stimulus of light, no sound makes any impression on the ear, or odor on the sense of smelling, the sense of taste is destroyed, the skin may be now seared with a red hot iron without the slightest indication of suffering; in fact, sensation, one of the great phenomena of animal life, appears to be annihilated. If we examine farther, we find that there is a total suspension of the intellectual functions, and that the patient is unconscious of any thing passing around him. If we go to the muscular system, we find that all that part of it which subserves to the purposes of animal life is completely paralyzed. The neck, trunk, and extremities have lost their power; and, if you raise the head, trunk, or one of the limbs, they fall down like dead masses as soon as the support is withdrawn. In some cases there is a certain degree of rigidity in the muscular system, in others not. We may observe, also, that from the paralysis of the buccinators, the cheeks are alternately puffed out and sucked in during respiration. As far as my experience goes, I believe that this symptom is fatal. Here, then, we see that the great phenomena of the life of relation are suspended. The functions of organic life, however, still continue to be performed, the heart beats, respiration goes on, and the power of secretion remains; but, after some time, the functions of organic life are also suspended, and the patient dies. In some of these cases, we observe evident signs of determination of blood to the head, the face is swollen, and the lips livid; there is considerable turgescence of the vessels of the neck, with heat of the head, the skin hot, and the pulse full and strong. In other cases, however, we have a feeble pulse and a cold collapsed state of the surface.

Let us now turn for a moment to the pathology of this form of the disease. I have already mentioned, that the extravasation sometimes occupies both hemispheres of the brain, or that it occurs on one side, by tearing through the substance of the brain, gets into the ventricles, and produces symptoms referable to a lesion of both sides. With respect to the simultaneous double effusion, the following is a short notice of some cases taken from the Clinique Medicale of M.

Andral. A man, about 37 years of age, fell down near La Charite in a fit of apoplexy. He was immediately brought into the hospital, had prompt and careful attention paid to him, but without any effect; he lay in a state of profound coma, with complete suspension of the phenomena of animal life, and died in an hour and a half. On examination there was a double effusion of blood found in the brain, but it had not got into the ventricles. In another case, marked by simple intensity, there was an enormous effusion discovered in the substance of one hemisphere, which burst into the ventricle, tore through the septum lucidum, and passed into the ventricle of the opposite side. In the next case, no distinct trace of optic thalamus or corpus striatum could be seen, their substance being completely broken up and destroyed by the effusion. I have told you that, after a rupture of the substance of the brain and the escape of the effused blood into the ventricles, persons have not recovered, but it is a fact, and a consolatory one indeed, that a person may recover from a *simultaneons double effusion.* A case in proof of this is given by Andral. A female, who had been for some time a patient at La Charite, died of cancer of the stomach. The history of her case was, that nine years before she had an attack of apoplexy. had fallen down in a state of insensibility, and remained comatose for a considerable time, that this was followed by *paralysis of both sides of the body*, which continued for two years, after which she gradually recovered the use of her limbs. In this case, two serous cysts, such as are met with in cases where patients have recovered from apoplectic attacks, were found, one in each hemisphere of the brain. In another case, the subject of which died of visceral disease, the patient had twenty-two years before an attack of apoplexy with double paralysis, and recovered with the loss of the use of one side; here there were two cysts also found. It appears, then, though extravasation, with rupture of the walls of the ventricles, and escape of blood into their cavities, always proves fatal, a recovery may take place after a simultaneous double effusion.

Let us now inquire briefly, whether an apoplectic attack, followed by paralysis of both sides of the body, gives sufficient grounds to enable us to make the diagnosis of either of these accidents. Does ir follow, if a person has an attack of apoplexy, succeeded by paralysis of both sides, that the effused blood has burst into the ventricles, or that a simultaneous double effusion has occured? Andral inclines to this opinion as far as I can collect. Dr. Abercrombie appears to differ from him, and gives cases in illustration of his opinions. The following is one:—A private of the 10th Hussars had been complaining for some time of a pain in the head, for which he was blistered, and the pain soon went off. On the 22d of July, 1819, he was seized with giddiness and fell down; on being raised, he vomited, and complained of violent head-ache and faintness, but was quite sensible. He was very pale, and his pulse slow and languid. He was brought into Hospital, where he asked for some cold water, made a few inspirations, and expired. From the moment of his last seizure he had been paralytic of both extremities. Here we have an attack resembling the first form of apoplexy, so far as complete loss of power in the upper and lower extremities is concerned; but observe, the *patient was not comatose and retained his faculties to the last.* On examination there was nothing found amiss with the brain, but, on removing the cerebellum, a coagulum to the amount of about two ounces was found under and surrounding the foramen magnum. Here the paralysis appears to have been produced by the pressure of the effused blood on the upper part of the spinal cord. This case is an interesting one. It appears that the injury done to the functions of the life of relation was partial, there was a lesion of the muscular function, but there was no coma, and the intellectual faculties were unimpaired. As far then as a single case goes, we may come to the conclusion, that we are not to make the diagnosis of the first form of apoplexy, unless, in addition to the double paralysis, there are coma and loss of intelligence and sensation. The great points of diagnosis are coma, suspension of the phenomena of the mind, and paralysis of both sides of the body, both of motion and sensation. We now come to consider the symptoms of the second or milder form of the disease. A person falls down in a state of insensibility, but, when you come to examine him, you find that the coma is not so profound, nor is the paralysis aud loss of sensation so complete. The eyes are to a certain degree susceptible of the impressions of light, signs of uneasiness are exhibited when strong pungent odors are applied to the nostrils, and indications of suffering are given if you pinch or burn the skin. All these circumstances prove, that the paralysis of sensation is by no means so complete in this as in the former case. You observe here, too, that instead of the cheeks being puffed out in the manner before described, there is only a partial paralysis of the muscles of the face, *and the mouth is drawn towards the sound side.* The patient, too, instead of dying in a comatose state, gradually regains his intelligence, and is only paralyzed on one side, or one extremity. All these circumstances point out that the injury done to the brain is not so extensive, and the occurrence of paralysis on one side shows that the effusion is limited to a single hemisphere of the brain. All this, too, is borne out by pathological anatomy, which shows us, in the first place, that the effusion is much less, that it exists only on one side of the brain, and never bursts into the ventricles. The general congestion of the head also is much less than in the former case. In the third form, the congestion and other symptoms are sometimes very slight. A person in health may feel a stunning sensation in the head, followed by some thickness of speech and drawing of the mouth to one side, or slight paralysis of one arm or hand, but he has no coma or loss of intelligence, and the paralysis quickly disappears. Every thing connected with the attack shows that it is very slight, the effusion is extremely limited, and this is confirmed by pathological anatomy.

I have now given you a brief sketch of the three varieties of apoplexy, between these you will meet with many intermediate cases.

Let us inquire how far does the circumstance of paralysis point out the occurrence of an extravasation of blood into the substance, or on the surface of the brain; that is, how far we can say that this patient has effusion, because he has become suddenly paralytic. It would appear, that the mere suddenness of the attack will not alone lead to the formation of a certain and accurate diagnosis. You will find in various authors many instances of affections of the head, not of an apoplectic character, in which there was sudden paralysis.

Thus, for instance, there are many cases of tumours and encysted abscesses on record in which there was sudden paralysis, and where, if you should pronounce the disease to be apoplexy, you would be certainly wrong. We had lately at the Meath Hospital, a remarkable instance of this. A patient, who had been for a considerable time laboring under aneurism of the innominata, in the course of the night became suddenly hemiplegic. On examining the brain post mortem, there was a circumscribed abscess found in one of the hemispheres, but no sanguineous effusion. If you look to the works of Abercrombie, Rostan, Lallemand, etc., you will find many cases detailed in which sudden paralysis occurred from other causes than apoplexy.

But are there no circumstances, which, combined with the suddenness of the attack, would lead us to form the diagnosis of apoplexy? Now it would appear that, as a diagnostic of apoplectic effusion, *suddenness of paralysis* is only to be relied on where there have been no premonitory symptoms of a local disease of the brain. In the great majority of cases of cerebral abscess, you will find that pains and cramps in some of the limbs, and pain of the head in the situation of the abscess, have preceded for some time the paralytic attack. But if a person in health, without any of these cramps or pains, gets a *sudden* attack of apoplexy, and becomes hemiplegic, you may make the diagnosis of apoplectic effusion with tolerable certainty. The fact of the paralysis, occurring with an apoplectic seizure, renders it highly probable that the case is really one of the hæmorrhagic diseases of the brain. On the other hand, it is true that we may have apoplectic effusions ushered in by symptoms of irritation of the brain; as in the case of an apoblectic effusion occurring in the centre of a softening of the brain. The absence, therefore, of these premonitory symptoms appears to be necessary towards forming the diagnosis of simple appoplectic effusion.

In cases where absorption of the clot takes place, we cannot suppose that any inflammatory condition of the brain exists; on the contrary, we have every reason to believe that a non-inflammatory condition of the brain is highly favourable to this process, for whenever any thing of an opposite character happens, we find that it prevents absorption. But sometimes cases occur, in which, at an earlier or later period, inflammation is set up round the clot. Now, what happens in many of these cases? Here let me repeat, that there are many exceptions to the rules given for forming the diagnosis of diseases of the brain; the variety in the symptoms of cerebral affections being so great, that it is sometimes difficult to deduce from them rules of general application. In most cases we have apoplexy followed by paralysis with resolution; but, in cases where inflammation takes place round the clot, it has been observed that the paralyzed limb which had been previously in a state of resolution becomes contracted, and then we have paralysis with contraction. This contraction generally comes on in a gradual manner, but, when the case is severe, it is frequently ushered in by violent spasmodic action of the affected limbs. We have, then, the following order of phenomena: first, paralysis with resolution, and then paralysis with contraction. In circumscribed inflammation of the brain, the phenomena are the reverse of these; we have first rigidity and contraction of the limbs, and then symptoms of apoplexy followed by paralysis with resolution. —*Ibid.*—*Ibid.*

13. *Treatment of Apoplexy.* By William Stokes, M. D.—I shall endeavor to get through the treatment of apoplexy as briefly as the important nature of the subject will admit. I shall commence by saying, in the words of Dr. Abercrombie, that the remedies for apoplexy are few and simple. The great point is to relieve the head from the accumulation of blood, to prevent farther congestion, and to obviate inflammatory action; and for these purposes the only efficient means we possess is bleeding. There is no disease in which the efficacy of free and bold depletion by the lancent is more remarkable than in apoplexy. I agree complely with Dr. Abercrombie in thinking that the symptoms which denote serous apoplexy by no means contra-indicate the use of the lancet; for I have already shown, that serous apoplexy was nothing but congestion, that the serous effusion was one of the consequences of this congestion, and by no means the cause of the apoplectic symptoms. Dr. Abercrombie thinks that, in the commencement of the disease you may bleed where the pulse is feeble as well as where it is strong and full, and gives many important cases in which the disease yielded to a copious abstraction of blood, though the state of the patient's pulse and general system at the time were such as would deter many from bleeding. He gives three cases of persons about seventy years of age, on whom this mode of treatment was practised with success, and another of a person of spare habit, aged eighty years, whose life was saved by a bold and timely use of the lancet. There is also another case detailed of a patient who was worn down and dropsical at the time of the attack, and received considerable relief from bleeding. I do not wish you to conclude from this, that you should bleed as boldly in the one case as in another; what I wish to impress on you is this, that in the vast majority of cases it is advisable to have recourse to the lancet. With respect to the first bleeding, I think that where the pulse is full and strong it should be large, and such as will produce some effect on the symptoms. This may be repeated afterwards to a smaller amount if necessary; but the subsequent bleedings should be rather local than general, except where there is any renewal of the cerebral and circulatory excitement, which must be always met with activity. I believe the cases in which you must make the largest bleedings are those in which there are symptoms of an hypertrophied heart. But where this is not present, one or two bold bleedings, followed by local depletion of the head, will be sufficient. In cases of apoplexy, you may either open a vein or the temporal artery, for the objections made to arteriotomy in phrenitis do not apply so much to cases of apoplexy. There is no violence on the part of the patient, nor is there the same chance of the vessel giving way. The head should be shaved and freely leeched, and the patient may be cupped on the temples or the back of the neck.

Next in efficacy to general and local bleeding seems to be the administration of strong purgatives. There are many cases on record, in which the coma and other symptoms have resisted bleeding, both general and local, but have disappeared under the influence of active purgation. One of the great objects in the treatment of apoplexy should be to get rid of the coma as soon as possible; and for this purpose nothing appears to answer better than the early use of brisk purgatives. Dr. Abercrombie recommends croton oil as the best purgative that can be employed, and indeed it is an excellent one; but if the patient can swallow, you need not be very anxious about the kind of purgative you prescribe; any active purgative followed by a strong enema will do. Where the patient cannot swallow, you may mix the dose of croton oil with some mucilage, and pass it into the œsophagus by means of a gum elastic tube.

After purgation, the next thing is to apply cold to the head by means of cold lotions, or iced water, or by pouring a stream of cold water on the head. This is a measure of great efficacy, and one which you may employ with safety and advantage.

In cases of apoplexy, where the coma had resisted free bleeding, both general and local, and where purgation and cold applications to the head have been employed without any decided effect, it seems avisable to apply a blister to the head or nape of the neck. You will recollect that I told you that blisters were always dangerous in the early periods of all acute visceral inflammations. This, however, does not apply so much to cases of hæmorrhagic effusion like apoplexy, in which blisters may be employed at an earlier period than in cases of active inflammation. I would advise you, therefore, to use blisters in cases of apoplexy attended by persistent coma, having first put into practice the means already mentioned.

Many persons advise the use of emetics in apoplexy, but the facts bearing on this point, to which I have drawn your attention when speaking of inflammation of the brain, will also apply here. You may take it as a general rule, that where congestion of the head exists, vomiting will always increase it, and must be, therefore, exceedingly dangerous. As far as theory goes it is totally against this practice, and I believe experience also is opposed to it. In a number of cases of disease of the brain, where emetics were employed, it has been found that an unfavorable result had ensued, and there are some cases of apoplexy on record in which the exciting cause was a fit of vomiting.

Suppose that, after having taken away blood, purged actively, used cold applications, and blistered the head, the coma still remains, accompanied by a feeble pulse and cold skin, what are you to do? I believe, under these circumstances and these alone, you may venture on the use of internal stimulants. Though this is at best but a forlorn hope, still the practice appears rational; we have analogy to guide us in the use of stimulants in such cases, and there are cases on record of persons who have recovered from this state by their judicious employment. The remedies most generally prescribed for this purpose are camphor, musk, and carbonate of ammonia. In the cases of typhus we know that these remedies have frequently succeeded in removing the coma; but I repeat that you should never have recourse to stimulants until the period for depletion has passed by, and all the ordinary means have failed.

I shall now suppose that we have succeeded in removing the coma, that consciousness has returned, and that nothing remains but paralysis of one side. Our great object is to get rid of the paralysis as soon as possible. Here you will recollect that you have to deal with paralysis depending upon extravasation, a paralysis which, as far as we know, will not disappear under any form of treatment until the extravasated blood has been absorbed. The first thing then you have to do, is to adopt measures to prevent a return of the attack. This is to be efected by carefullyrestricting the patient in his diet, by avoiding all causes of cerebral irritation, whether physical or moral, and by obviating every thing capable of exciting the circulation. But you should not be content with this; you should, from time to time, employ local depletion, which in cases of this kind has a double utility. It tends to prevent a repetition of the attack, and, by lowering the circulation, keeps the brain in that non-inflammatory condition, which is most favourable towards promoting the absorption of the coagulum. In many cases, also, you will find it of great advantage to establish a drain in the vicinity of the disease, and a great deal of good may be done by putting a seton, or an issue, in the neck. You must also pay constant attention to the state of the bowels and urinary system in cases of paralysis; keeping up a steady but mild action of the bowels has an excellent effect, and I need not impress upon you the necessity of paying strict attention to the bladder. —*Ibid.*—*Ibid.*

14. *Paralysis consequent on Apolexy.* By William Stokes, M. D. With respect to the paralysis which is consequent on an attack of apoplexy, there is the greatest possible variety. In some cases there seems to be paralysis of all, or almost all, of the muscles of animal life; in others, it affects only the muscles of one side of the body. A rare and extraordinary form of paralysis has been described by the French writers, who have given it the name of *paralysis croissee.* In this form of the disease, there is an affection of both sides, but not of the symmetrical members; for we find the left arm and the right leg paralyzed, and *vice versa.* This is an unusual form, in fact, the rarest to be met with in practice. We may also have great varieties in the amount of the paralysis; in some cases both sides being affected, in others only one, while in others there is only a single extremity or one side of the face paralysed. We may also have complete paralysis of one side without any affection of the face. I remember a remarkable case of this kind, of which I shall give you an abstract. A gentleman, of stout muscular habit, and with a strong full pulse, had been suffering for a long time under an obstinate gouty affection. From a repetition of the gouty attacks he got a chronic swelled state of the lower extremities, which continued for some time, he being in other respects in the enjoyment of excellent health. The swelling, however, prevent ng him from taking his usual exercise, he applied for advice. Laced stockings were advised, the effect of which was, that the œdema had subsided, and the motion of the lewer extremities was restored. It is curious, that, between the period of the removal of the œdema and the paralytic attack which I am about to describe, this gentleman enjoyed excellent health. At the end of that time, on attempting to go over a step that led into the yard, he found he could not accomplish his purpose, and struck his foot against the stone. He immediately became alarmed and sat down, and soon after found that he had lost the power of using his arm. I saw him in a short time after the accident, and found that there was complete paralysis of the arm and leg, but no distortion of the face or tongue, or the slightest lesion of intelligence. He continued in this state for some time, and then recovered, but it was necessary to take a large quantity of blood from him. In the first bleeding, as the pulse was full and bounding, I took sixty ounces of blood from the arm, and I think it was owing to the activity of the measures adopted, that he recovered so speedily. I mention the case merely to show that we may have paralysis of the leg and arm, without any affection of the face, or loss of intelligence. In some cases we find the paralysis affecting the tongue, face, and muscles of the eye-lids; in some we have paralysis of the sphincter ani, or of the muscles of deglutition, or of the bladder, but these are rare, and the most ordinary form is paralysis of the muscles of one side, and distortion of the face. There is another circumstance, which seems to be of so frequent occurrence as to form a law, perhaps the most general of any in medicine, that paralysis occurs on the side of the body which is opposite to that on which the effusion occurs. If you have an effusion into the right hemisphere, you will have paralysis of the left side of the body, and, if the effusion be on the left side, the paralysis will be on the right. To this rule, however, it has been stated that there have been a few exceptions; how they have occurred it is totally impossible to explain, it is sufficient for us to know that such exceptions have been witnessed. Cases of this description have been very rarely seen since pathological anatomy has been studied with more diligence; it is, however, true, that a few facts have been detailed by men of great professional eminence. We want facts to throw light on this point, and, until this is accomplished, we must remain in ignorance of the cause of the anomaly. In the vast majority of instances, the paralysis is on the opposite side to that on which the effusion takes place, and this appears to be explained by the decussation of the fibres of the brain at the upper part of the spinal marrow, the fibres of the left side passing to the right, and *vice versa*. It is an interesting fact connected with this subject, that the muscles of the face follow the same law as the muscles of the extremities, and yet it is a fact, as you are well aware, that the nerves which supply the muscles of the face come off before the decussation of the fibres of the brain takes place. The fifth nerve, which supplies the face with branches, is given off at a considerable distance from the decussation of these fibres, and yet we perceive that the muscles, to which it is distributed, obey the same law as those which derive their nerves from the spinal cord. Now, if this decussation was the only cause of the paralytic symptoms being observed on the side opposite to that in which the effusion occurs, the muscles of the face should be an exception to this law, but we find that they correspond with other parts of the muscular system in this respect. Thus if a man gets an attack of apoplexy, followed by paralysis of the left arm, we find the left side of the face affected, and *vice versa*. We must conclude from this, that the mere decussation of the fibres is not the sole cause of this peculiarity, and must look for an explanation elsewhere, by referring it to the intimate communication which exists between both sides of the brain by means of its commissures. Many persons are not familiar with the phenomena of the face and tongue in paralysis; they are, however, simple and easily explained. Let this diagram represent the head—here we have the right hemisphere of the brain, here the left. Now, suppose you have an apoplectic effusion in the right hemisphere, the consequence is, that you have paralysis of the left side of the body, according to the law already mentioned. What will then happen with respect to the fact is, that the muscles of the left side being paralyzed, and their antagonism being destroyed, the mouth is drawn by the sound muscles of the opposite side from the paralyzed side, and this is invariably the case. Recollect then that the mouth is always drawn from the paralyzed side, and towards that side where the disease exists in the brain. But when you desire the patient to put out his tongue, do you find that the tongue follows the direction of the mouth? No; it goes towards the opposite side. This appears somewhat paradoxical at first, but is easily explained. The protrusion of the tongue is effected by the action of the genio-hyo-glossi muscles, which are, as you all know, a pair of fan-shaped muscles, attached to the inside of the chin, the middle line of the tongue, and the body of the os hyoides. This diagram will represent it. Here is the muscle of the left side, and here is the right. When the patient puts out his tongue, this left half being paralyzed, and having lost its antagonism, the tongue obeys the action of this, the right half, and the fixed point of attachment of the muscle being to the right of the mesial line, the base of the tongue is brought forward, and to the right, and its point consequently deviates to the left or paralyzed side. It has been remarked also, that there is some variety with respect to the paralysis of the tongue; some patients can protrude it, others cannot. In some cases too, the patient can put out his tongue well enough, but he cannot employ it in the articulation of sounds, and his speech is quite indistinct.

With respect to paralysis of the extremities, the upper are paralyzed more frequently than the lower; and, when both extremities are engaged, the upper are generally more completely affected than the lower. When a person recovers, also, we find that the lower extremities are the first to regain their lost power and sensibility. These circumstances have been attempted to be explained by considering the particular parts of the brain in which the effusion has occurred; but, as this has not as yet been sufficiently made out, I shall pass it over. I regret, also, that I have not time to enter into the subject of the different varieties of lesion of intelligence in cases of apoplexy. I must, however, observe, that the varieties are infinite, and your trouble will be amply repaid by reading what has been written on this point by Dr. Abercrombie, and Dr. Cooke in his Treatise on Nervous Diseases. You will find in the latter work an extraordinary collection of facts with respect to lesions of the intellectual functions.—*Ibid.*—*Ibid.*

15. *Paralysis from disease of the Arterial System.* By William Stokes, M. D.—I wish to call your attention to a remarkable form of paralysis, in which the disease appears, as far as we can see, not to depend on any primary lesion of the *nervous system.* In this form we have a paralysis, not the result of any disease of the brain or nerves, but connected with an affection of the vessels of the part. This is a very singular disease, and I am anxious you should be acquainted with it, for I believe it is by no means so rare as many persons think. The other point to which I would direct your attention refers to the influence of magnetism on the human body; of this I shall speak on a future occasion, confining myself for the present to that form of paralysis which is connected with disease of the *vascular system.*

So as to give you some idea of this affection I think I cannot do better than read for you the notes of a case of it, published by Dr. Graves and myself in the fifth volume of the Dublin Hospital Reports.

A man, aged 44 years, was attacked in December, 1828, with alternating sensations of cold and burning heat in the toes of the right foot. These extended to the leg, of which the power became diminished. Pains in the foot next occurred, and in a month the part became cold and wholly deprived of sensation.

On the day of his admission the pain suddenly extended to the calf of the leg; and from this time he lost all power of motion in the leg. On admission the temperature of the body, with the exception of the affected limb, was natural. The pain had extended to the thigh during the night. The temperature of the limb was but 58 deg. of Fahrenheit. Slight œdema existed about the ankle. There was complete loss of sensation from the middle of the thigh to the toes; the patient could rotate the thigh slightly, but there was no other voluntary motion possible. The femoral artery appeared like a hard cord, painful on pressure, and without pulsation. By the stethoscope we found that pulsation was also wanting in the common iliac on this side, while that of the left iliac was plainly perceptible. The patient died on the fourth day after admission, the limb having become purple, tender, and covered with vesications.

On dissection, the right common iliac appeared distended and livid, and was completely plugged up by a dark clot, extending to the external and internal iliacs, and engaging the gluteal and obturator arteries. The same occurred in the femoral and profunda, and extended as far as they could be traced, to the tibial arteries, and to the peroneal. The lining membrane of these vessels was soft, villous, and red; the clot in some places being separated from it by a layer of puriform matter. No disease in the veins. A large portion of the vasti and rectus muscles was *white* and hardened. Here you perceive a train of symptoms, some of which might be referred to disease of the brain, if the man had any cerebral symptoms, which was not the case, for his intellect was sound, and he had no evidence of cerebral disease except the paralysis.

His constitutional symptoms were emaciation, prostration of strength, and loss of appetite. The temperature of the body was natural, but, on examining the limb, we found, (and this is a point of great importance,) that it was as low as 58 deg. of Fahrenheit; in fact, it was quite cold. There was also complete loss of sensation from the middle of the thigh to the toes, and though he could rotate the limb slightly, it was, in all other respects, powerless. Here we have paralysis of motion and seasation in one of the extremities, *with remarkable coldness of the limb.* On making an examination along the track of the femoral artery, we found that it was painful on pressure, *without any pulsation*, and conveying to the finger the feel of a piece of hard cord. From a consideration of those circumstances, we came to the conclusion that it was not pervious, and that this would account for the state of the limb. In this case, also, we made another remark, and this, I believe, is the only instance on record in which such a diagnosis was made. Up as high as the groin, the pulsation of the femoral artery could not be felt, and we were anxious to ascertain how far farther the disease extended. The state of the femoral artery in the left groin was natural. On making an examination with the stethoscope, we found that the pulsations of the aorta were perceptible down to its bifurcation, but when the stethoscope was applied below this on either side, we observed that there was no pulsation in the right common iliac artery, but on the left side it could be traced distinctly down to the groin. Here then we had a train of phenomena, such as ordinarily occur in paralysis affecting the right lower extremity, and along with this an obstruction to the circulation in the thigh and leg. From these circumstances we made the diagnosis of obstruction of the right iliac and femoral arteries. On dissection we found that the aorta was healthy to within about six inches of its bifurcation; below this point it was partly filled by a red clot. The left common iliac was healthy, but the right was plugged up with a dark red clot, which extended into the internal iliac and obturator arteries, filling up also the femoral and its branches. The case, in fact, was nothing more nor less than one of chronic arteritis.

This remarkable form of disease has been also observed by other authors. You will see in Rostan's work on the softening of the brain, the reports of two cases of this disease, occurring in patients of extremely advanced age. In one, there was complete paralysis of the right arm, which was cold and livid. The fingers were threatened with gangrene, and no pulsation could be felt in the radial artery. By stimulating frictions a certain degree of warmth and motion was restored, and it was even thought that pulsation could be perceived. By degrees the power of the left arm of the lower extremities began to fail, with diminution of the force of the pulsation. On dissection, extensive disease of the arteries was found; the right brachial, at the insertion of the deltoid, was obliterated by a mass of fibrin, below which the vessel was contracted and closed; the left brachial artery was also narrowed, but without any clot; and this condition was farther met with in the crural vessels. The cerebral arteries and the aorta was diseased. In the second case, the patient, aged eighty, was attacked with violent pains in the left leg, which became cold and bluish. There was no lesion of intelligence, and the corresponding arm was unaffected. In fifteen days, the pains having augmented, a certain degree of paralysis supervened, which, however, was never complete. On dissection, (the disease having lasted a month,) the crural artery was found extensively obliterated by a fibrinous clot. Here you observe, gentlemen, that, notwithstanding the great age of both patients, the disease was not *ossification*, but, in all probability, *arteritis*.

In persons advanced in life, the arteries are also frequently obstructed by the formation of ossific deposits within them, producing loss of power, coldness, and diminution of sensation. A similar fact may occur from the pressure of an adjoining tumor on the trunk of a principal artery.—*Ibid, Sept. 6th and 13th*, 1834.—*Ibid.*

13. *Diagnosis of Paralysis from disease of the Arterial System.*—By William Stokes, M. D.—Paralysis resulting from disease of the arterial system, is distinguished from paralysis caused by cerebral disease, by the following marks: first, by the color of the integuments of the affected limb, which, in a case of the former description, are generally of a violet hue, or of a much deeper tinge than in the latter case, or in a state of health. It is very rare to find the two limbs of the same color, as we do in cases of cerebral paralysis. Another mark is, that the temperature of the limb is always lower than that of the healthy one; but the distinctive sign of this form of paralysis *is the absence of pulsation in the arteries in parts where it should be naturally observed.* If to this description you join the absence of cerebral symptoms, you will seldom fail in making a correct diagnosis. I have had two cases of this disease under my care; one of them occurred in the upper, the other in the lower extremity, and, from observing the characteristic marks already detailed, I had no difficulty in making the diagnosis.

The subject of this last case is a gentleman who has been for the last four of five years laboring under paralysis of the lower extremities unaccompanied by any symptoms indicating disease of the brain. His intellect remains not only unimpaired, but in a state of high activity; and, what is equally singular, he has had none of the usual symptoms of disease of the vertebræ or spinal cord. His limbs, however, are quite powerless, and are of an *icy coldness*, and yet you will hardly believe me when I tell you that I have repeatedly felt the femoral, popliteal, and even the anterior tibial arteries pulsating distinctly! This is a singular fact, but I have verified it by a number of observations. You will perceive, then, that in taking a remarkably diminished temperature as a diagnostic of paralysis from arterial obstruction, we must admit that as a sign it is only valuable *when combined with absence of arterial pulsation.* In this case, the fact of such extreme coldness of the lower extremities at the same time that their circulation continues with undiminished activity, becomes of great importance, as tending to prove that the temperature of the body depends more upon the state of innervation than on arterial action. There are, indeed, many facts which go to prove that animal heat is more closely connected with the nervous system than with the circulating.

It is to that peculiar form of this disease, which is considered by some authors to depend on ossification of the arteries, that the name of "Pott's Gangrene" has been applied. A great deal of light has been thrown on this disease by the researches of modern pathology. It is now pretty well established, that we may have this gangrene, not only in old persons from ossification of the arteries, but also in the young from arteritis. In truth, the pathology of Pott's gangrene appears to be of one or two changes,—either an arteritis or ossification of arteries themselves; and of these two causes the first is by far the most frequent. You will see at once the importance of this view of the question, for if the gangrene occurs in a young person, and is connected with inflammation of the arteries, it is a disease more or less under the control of medical treatment; but if it be produced by ossification of the arteries, the results of treatment are far less likely to be successful.

We have, then, in a case of paralysis of this description, more or less loss of sensation and motion, coldness of the limb, and absence of arterial pulsation. With respect to coldness, it may be said that it is of little value as a sign, being frequently observed in cases of cerebral paralysis. To this it may be replied, that though colness is sometimes present in cases of ordinary paralysis, still it is never so remarkable as in this form of the disease, and the temperature of the limb is but a few degrees below the standard of health. Dr. Abercrombie makes a very interesting conjecture on this subject. He says, the temperature of paralyzed limbs is generally considered to be lower than that of the healthy ones, and, indeed, such is the case; but the true explanation of this occurrence is, that in this condition the limb loses its power of preserving a medium temperature, and hence it is that, according to the temperature to which it has been exposed, it becomes hotter or colder than the healthy limb. A case is mentioned of a medical man who labored under paralysis of one of the upper extremities. This gentleman, on one occasion, after having applied some warm bran to the paralyzed limb, was astonished to find, on touching it with his sound hand, that he could not bear the heat, though he was at the same time unconscious of any increase of temperature in the paralytic extremity.

The symptoms, then, of this form of paralysis, are diminution or abolition of sensation and the power of motion, a dark or violet hue of the skin, remarkable coldness, and absence of pulsation in the arterial trunks which supply the affected limb. These, with a tendency to the formation of gangrene, are the characteristic marks of the disease, and, by bearing them in mind, you will seldom err in making a diagnosis. In the great majority of cases the disease is confined to one extremity; but Rostan gives some cases in which it was more general. We might also add to the diagnosis, that paralysis connected with disease of the brain often comes on suddenly, while, in this case, its invasion is slow and gradual. It is, however, true, that some cases of paralysis, depending on this cause, have come on so suddenly, as to render this circumstance of less value as a diagnostic. —*Ibid.*—*Ibid.*

14. *On Paraplegia.* By William Stokes, M. D.—Before I leave the subject of paralysis, I wish to draw your attention to one more form of the disease by no means uncommon; I allude to that in which *both* the lower extremities are exclusively engaged. This is a disease or symptom which may arise from a great number of causes, and be observed under a variety of circumstances. Generally speaking, however, it will, in almost every instance, be found to depend on some cause which engages the spinal marrow, either primarily or secondarily. I believe that this paraplegia, as the result of disease of the brain, is never met with except in combination with paralysis of the upper extremities. General paralysis may be produced by cerebral disease, and in describing the various forms of paralysis which depend on disease of the brain, this form has been particularly noticed, but when paralysis of the lower extremities *alone* occurs, it is generally the result of some lesion of the spinal marrow, either organic or functional, below the situation in which the brachial nerves are given off. Among the causes by which this paraplegia is produced, the following are the principal: inflammations of the membranous coverings of the spinal cord with effusion of lymph or serum; spinal apoplexy; ramollissement from inflammation of its substance; pressure on the cord, by solid tumors from a variety of causes; the bursting of abscesses or aneurismal swellings into the vertebral canal as occurs in some cases of aneurism of the abdominal aorta. Thus during the progress of a case of this description, it has been observed that the patient suddenly became paraplegic, and, on examination after death, a quantity of blood which escaped from the aneurismal tumor has been found compressing the spinal marrow. Lastly, recent investigations have established the fact that we may have paralysis of the lower extremities, and yet on dissection *we cannot detect any traces of disease in the bones of the vertebral canal, or in the membranes or substance of the spinal cord.* Hence, gentlemen, you see how cautious you should be in making the diagnosis, so common among surgeons, of caries of the vertebræ, in cases of paralysis of the lower extremities. The truth is, that in the present state of medicine on this subject, we labor under very great difficulties; the diagnosis of these affections is exceedingly obscure; it is a subject still open to investigation, and I need not remark that it is one of paramount importance.

Paraplegia is one of the most miserable diseases to which the human body is liable. It is almost always obstinate and unmanageable, and in the majority of cases incurable. How far the fatality of the disease depends upon the want of an accurate diagnosis, and a correct plan of treatment, must be determined by future observations, but it is a fact that a vast proportion of paraplegic patients die, and under the most melancholy circumstances. In many cases the formation of gangrenous sores on the back and loins is a common occurrence. For this there are two reasons; first, the vessels of those parts exposed to pressure from position fall into that state which Andral terms *mechanical hyperæmia,* the result of which is that they are unable to unload themselves, a stasis of blood follows, and this leads to mortification of the part; secondly, there is a lesion of inervation. Hence it is that the great majority of patients of this kind die with gangrenous sores on the back and loins. They have also most constantly paralysis of the bladder or its sphincter, or both, producing retention of urine, or retention with incontinence, or stillicidium urinæ. The sphincter ani, too, is generally paralyzed, and we have a most melancholy and disgusting source of annoyance. The frequent passing of urine and fæces keeps the unfortunate sufferer in a state at once pitiable and loathsome, and when, in addition to his other calamities, the gangrenous sores form, the supervention of low diffused erysipelatous inflammation may prove fatal; or he may be carried off with symptoms of typhus fever from the absorption of putrid matter.

While on the subject of paraplegia, I am anxious to lay before you a sketch of some important opinions lately put forward by Mr. Stanley of London. In the last number of the Medico-Chirurgical Transactions, this gentlemau has written a most interesting paper, in which he gives the history of several cases of paraplegia, the majority of which were supposed to be examples of caries of the vertebræ, but in which, on dissection, no disease could be discovered, either in the bones of the vertebral canal, or in the membranes or substance of the spinal cord. You will ask, were there no pathological phenomena in these cases? There were, but they belonged not to the spine or its contents, but to an organ in its immediate vicinity—the kidney. From a candid review of Mr. Stanley's cases, there appears to be reason to believe that disease of the kidneys may produce all those symptoms which have been attributed to lesions of the spinal marrow, or caries of the vertebræ. In the four first cases, the symptoms given as of caries of the vertebræ were present, and the cases treated as such. On dissection, no caries, or disease of the cord, could be discovered in any of them, but the kidneys were found to be the seat of extensive disease. The fifth case was a remarkable one: the patient had been admitted for retention of urine, the consequence of severe gonorrhœa, which had been checked by injections. The bladder and sphincter ani became paralytic, and he lost the power of the lower extremities to a certain degree. He also complained of severe pain at the fifth lumber vertebra. He distinctly traced the pain from the bladder to the left kidney, and then to the right. Paralysis of motion, and, nearly, completely of sensation of the lower limbs, next supervened, and in about a fortnight he died. On dissection, the kidneys were found in a state of inflammatory softening, and with numerous depositions of pus. The bladder was inflamed, but the brain and spinal cord were perfectly healthy. In the sixth case, a patient, while in progress of cure of a gonorrhœa with phymosis, was suddenly seized with paraplegia. The functions of the brain were unaffected. He had suffered for a day or two from pain in the loins. Sixteen hours after this attack, he suddenly died.

From considering the former cases, Mr. Stanley predicted that inflammation would be found in the kidneys. A slight turgescence of the vessels of the cord, with a little transparent effusion in the theca, were found, but the kidneys were in a state of the most intense engorgement. In this case, it was remarkable that from the period of the paraplegia, there was no inordinate secretion of urine. The seventh case was that of a patient who, for two years, had been laboring under pain of the back, increased by pressure, and incontinence of urine. On dissection there was some vascularity and effusion of the cord, but both the kidneys were almost entirely destroyed by disease. In addition to these, Mr. Stanley mentions four more cases, which were seen by a friend of his, Mr. Hunt, of Dartmouth, which corroberate his opinions.

Here, then, we may have well-marked paraplegia without any perceptible organic change in the spinal cord or its investments, but presenting distinct traces of disease of the kidneys. This leads us to observe the very close connection which exists between the kidneys and spinal cord, a connexion which has been long recognized by medical practitioners, but only in a limited point of view, for though they were of opinion that disease of the kidneys, and a discharge of ammoniacal urine, were the results of spinal disease, they never seem to have reflected that the reverse of this might happen. It seems now, however, to be almost completely established, that disease of the kidneys may produce symptoms which are referable to disease of the spine; and Mr. Stanley has the credit of having been the first who directed the attention of the profession to this circumstance, and his paper must be considered as one of the most important, and practically useful, which has appeared for a length of time. You should all peruse it carefully. The fact that disease of the spine will give rise to affections of the kidney, is long known, and has been proved by numerous experiments. Thus Ollivier details the experiments of a German physiologist, M. Kreimer, who, by dividing the lower part of the spinal cord in animals, made the urine ammoniacal. You will also find in Dr. Prout's work, that an ammoniacal state of the urine may be rapidly brought on by injuries of the back from falls or bruises on the spine. It is indeed singular how quickly those profound functional lesions of the kidneys supervene on injuries of the spine, sometimes appearing in four or five days, sometimes sooner. Medical men have hitherto been in the habit of looking at this matter only in one point of view; they know that disease of the spine will produce disease of the kidneys, and here they stop; but it has been shown that the reverse of this may happen, and that renal disease may produce very remarkable lesions in the functions of the spine. Of this very curious occurrence we have many analogies in pathology. Thus, for instance, in several cases of cerebral disease, but chiefly in hydrocephalus, we have vomiting; here we have functional disease of the stomach depending on an affection of the brain. Take the reverse of this,—observe the delirium which attends a case of gastro-enteritis,—here you have the functions of the brain deranged in a most remarkable manner, and this produced by sympathy with an inflamed mucous membrane. The truth is, that in the spine and kidney, as well as in various other parts of the system, we have two organs which are so closely connected by sympathy, that disease of one will bring on serious functional lesion of the other.

Observe then, gentlemen, the great importance of these inquiries. When you meet with a case of paraplegia, you are not at once to conclude that it depends on disease of the spine or caries of the vertebræ. You must carefully investigate its history, and ascertain whether it may be referred to either of these causes, or whether it may not rather depend on disease of the kidneys. That it may depend on the latter cause is now established, for the cases are too numerous for us to suppose the complication accidental. You will observe the importance of making an accurate diagnosis when you consider that this point will most materially influence your treatment. In the one case, your treatment will be directed to the bones and cartilages of the spine; in the next, to the spinal cord itself; and, lastly, to the kidney, a parenchymatous organ, to which there is a great determination of blood. No one will venture to assert that the principles of treatment in each of these cases are the same; and the chances are, that, if you do not make a correct diagnosis, you will practise improperly, and without success. I have now seen a number of these cases, but there were only two of this dsecription in which I was fortunate enough to obtain a post mortem examination. I cannot say that my dissection exhibited remarkable disease of the kidneys, (they were large and very vascular,) but, from the many points of resemblance they bore to Mr. Stanley's cases, I was led to conclude, that if they were not examples of actual chronic disease of the kidney, they were cases of lesion of function in the spine, unaccompanied by any organic change to account for the symptoms. I shall briefly detail these cases;—the first was that of an unfortunate man from the country, who was discovered by two friends of mine under peculiar circumstances. While on an excursion they were requested to visit a poor man who was lying ill at a remote farm-house. They heard he had been laboring under a dropsical affection for a long time, and had been treated for ascites. On arriving at the cottage, they found the man lying in bed, with his abdomen very much enlarged; and, on farther investigation, discovered that he was quite paralytic of the lower extremities. On examining the belly more particularly, they found that the swelling was produced, not by ascites, but by *an enormously distended bladder.* He had also stillicidium urinæ, with paralysis of the bladder; and this having been mistaken by the medical practitioner who attended him for suppression of urine he had prescribed diuretics, and continued this plan of treatment for some weeks, totally overlooking the paralysis of the bladder. As little or nothing could be done for him in the remote situation in which he lived, it was determined to send him up by easy stages to Dublin, and procure him admission at one of the public hospitals. On his arrival he was received at the Meath Hospital; and when I visited the wards next day, I found that he was quite paralytic of both lower extremities, that the bladder was in the state above described, and that his health had suffered considerably, and that bed-sores had formed on his back, and were increased by his journey. I prescribed cupping and blistering, which were productive of same slight relief, but in the space of a few days he began to exhibit symptoms of low typhus, as if from the absorption of pus, and sank rapidly. On examining his body, we could not detect any traces of disease in the bones or cartilages of the spine; neither did the cord, or its membranes, present any marks of organic lesion, except that towards its lower portion, where it begins to spread out into the cauda equina, it was perhaps a little softer than natural. I regret very much that I did not note the circumstances of this case more fully; but, as far as my recollection of it goes, the general features were as I have just mentioned. I had another case some time since in the Meath Hospital, in which the following circumstances were observed:—The patient, a laboring man, generally employed about the quays, was brought into the hospital with paraplegia of some standing. The first symptom in his case belonged not to the spine, but to the urinary system; he had an attack of gonorrhœa, for which he had used stimulants and balsams; and, in some weeks after, without any injury to the spine, he lost the use of his lower extremities. During his stay in the hospital the urine was intensely ammoniacal. On examining his body after death, we could not discover any disease of the bones or spinal marrow. A layer of substance resembling fat or organized lymph was found lying on the theca of the spinal marrow, but it was so very small as to be scarcely sufficient to account for the symptoms. The kidneys were pale, flabby, and without any vascularity, but did not present any marked traces of organic lesion.

Here then, gentlemen, were two cases which, before the publication of Mr. Stanley's paper, would be considered as examples of organic disease of the spinal cord or its investments; and yet, on dissection, we can find nothing to establish this opinion; and, in the last one, the first affection was of the urinary system.

Is it possible that a *functional* disease of the urinary system may produce also a *functional* disease of the spinal cord?

With respect to the diagnosis of caries of the spine, I wish to make a few observations. The diagnosis where there is distortion of the spine, is extremely easy, but this does not hold where the caries is accompanied by distortion. Let us inquire—are there any circumstances which would enable us to arrive at the diagnosis of caries without distortion? One symptom, not observed, as far as I can see, in paralysis *connected with disease of the kidney* is, that the patient feels exquisite pain on motion. This is an exceedingly common symptom in caries of the vertebræ, but I am not aware that it occurs in cases where the disease is situated in the kidney or the spinal cord itself. There is another remarkable circumstance:—When the patient attempts to move, he often feels a cracking sensation in the affected portion of the spine; and this has not only been observed by the patient himself, but is also perceptible to his medical attendants. When this occurs, it may, I think, be looked upon as a diagnostic symptom. The exquisite pain on motion, the tenderness of the spine on pressure, and the cracking sensation,—these might be sufficient to make the diagnosis of caries of the vertebræ, even in cases where there was no distortion. But if you had a case of paralysis of motion and sensation of the lower extremities, and if these symptoms came on without any injury of the spine, if there was little or no tenderness on pressure, if the patient felt scarcely any pain on turning or moving, and if he had at the same time symptoms of disease of the kidney or bladder, and ammoniacal urine, under these circumstances the great probability would be, that it was not a case of caries of the vertebræ, or original disease of the spinal cord or its investments, but a lesion of function of the spine, connected with organic or functional derangement of the kidneys. It must be acknowledged, however, that the diagnosis of this affection is rather obscure. The circumstances which I have just mentioned might enable you to get rid of the opinion that it was caries of the vertebræ or organic disease of the spinal cord, and that it was probably such a case as Mr. Stanley has described; and if you could arrive at this diagnosis at an early period of the case, it would be a matter of great importance. By doing this you would then be aware that you had to deal with an inflammatory affection of a highly vascular organ; you would not be led away from the real state of the case, or waste time in treatment calculated to stimulate the spine, or remove disease of the vertebræ. Your plan would be simple, and your treatment defined, and all your efforts would be directed towards removing the disease of the kidneys. You will easily perceive, gentlemen, that diagnosis is here of vast importance; unfortunately, it is still involved in obscurity.

The prognosis of cases of paraplegia, when once complete paralysis is established, should be always unfavorable. The fact of paralysis occurring, is sufficient in itself to prove the existence of extensive disease in most cases. There may be, however, some cases susceptible of cure, and this particularly occurs in young females, in whom a perfect cure has been frequently accomplished by the use of stimulent embrocations to the loins. I have seen one case of this kind, in which the patient was paraplegic for a year and a half, cured by the application of hot oil of turpentine over the lower part of the spine. Simple as the treatment may appear in this case, its success was rapid and complete. Mr. Crampton has mentioned to me another case, in which the patient's limbs were quite rigid, and could not be moved without great difficulty; in this case, complete relief was obtained by applying Pearson's liniment over the lower part of the spine. This liniment produced powerful counterirritation, and an eruption of bullæ over the body, which were speedily followed by relief. The patient is now in the enjoyment of good health, and since the period of her cure, which is now better than six years ago, has had no return of the disease.—*Ibid, Sept.* 13, 1834.—*Ibid.*

18. *Treatment of Paralysis consequent on Apoplexy.* By WILLIAM STOKES, M. D.—The paralysis, which supervenes on an attack of apoplexy, is to be treated always in the first place by means directed to the head, and the brain is to be put in such a state as will favor the removal of the clot by the means already recommended; in addition to which it will be necessary that the body and extremities should be kept in a warm temperature. But there is this very singular circumstance connected with some cases of paralysis, that a period will arrive when, although the original disease of the brain has been removed, and the clot absorbed, the paralysis still continues. It is not easy to explain this circumstance, but it has been observed in many persons, who have been paralytic, that the clot was completely absorbed, and no existing trace of disease discoverable, such as would account for the continuance of the paralysis. In cases like this, we must adopt a different mode of practice, and have recourse to measures capable of exciting the brain, and we have reason to believe, that whatever will excite the brain and restore its energy, (I must use this phrase for want of a better,) will cure the paralysis. We find that, in some cases, where the brain of a patient, under such circumstances, has been exposed to any sudden stimulus, whether physical or moral, the symptoms of paralysis have disappeared, sometimes gradually and slowly, at other times rapidly and at once. Now, this disappearance of the symptoms shows that the paralysis did not then depend on the presence of a clot, for if an unabsorbed coagulum remained in the situation of the original extravasation, the paralysis would not disappear. But it has been frequently observed, that a patient, laboring under paralysis, may get rid of his symptoms suddenly, or that, at a certain period, they begin to decline, and then go away altogether. From a consideration of these circumstances we are led to divide the treatment of paralysis of this description into two parts, and endeavor first to excite the brain itself and next the nerves, which supply the paralyzed limbs. For this purpose several remedies, supposed to be capable of stimulating the brain, so far as its action on the muscular system is concerned, have been recommended, the most important of which is the nux vomica, or its active principle, strychnine. The researches and experiments of modern medicine have already established the efficacy of strychnine in such cases, but you will recollect, as I before stated, that this powerful remedy can be employed with safety only in cases where the paralysis continues after the disappearance of organic disease of the brain. Until that period arrives, and all symptoms of congestion and excitement are removed, it would be improper to prescribe the use of strychnine. One of the most recent publications on this subject is from the pen of Dr. Bardsley, of Manchester, in which you will find an exceedingly interesting series of cases treated with strychnine, and many of them with the most decided success. In most of these cases, you will find that Dr. Bardsley, even where the disease has been of some standing, *precedes the use of strychnine by measures calculated to deplete the head*, even though the cases were chronic. Hence, whenever you are about to prescribe this remedy, you should be satisfied that depletion has been sufficiently performed. You may be called to treat a patient for paralysis after an apoplectic attack. Here you must consider how far you are to premise the use of strychnine by depleting measures, and you must also reflect that we here have shadowed out one of the most important principles in medicine, *that in almost all cases where a cure is to be attained by stimulation, it will be effected more readily, and with much more certainty, when preceded by local depletion, no matter how long the disease may have lasted*. The efficacy of strychnine in paralysis seems to be dependent on the antecedence of local or general depletion.

Strychnine being an exceedingly active remedy, and having a most powerful effect in stimulating the brain, it being also, one of the accumulative class of medicines, it will be proper to commence its exhibition with a very small dose, and watch its effects with care. The following is the formula which I would recommend you to employ. You take a grain of strychnine, and your object being to divide it into a number of equal parts, (say sixteen,) to ensure an accurate division, you dissolve it in a small quantity of alcohol, and having mixed this solution with a sufficient quantity of bread crumb or conserve of roses, you divide it carefully into sixteen equal pills. In this way you may be tolerably certain that each pill contains one-sixteenth of a grain. Begin at first with one pill a day, next day you may give two, and so on until you have brought it up to half a grain or a grain, watching carefully its effects. Now, what are these effects? They are very analogous to the phenomena produced by inflammation of the brain taking place in the vicinity of the clot; namely, spasms of the muscular system.

It is also a curious fact, that these spasms are principally observed on the paralyzed side; in other words, that the portion of the brain which has been affected by disease is more sensible to the stimulus of the strychnine, the consequence of which is spasmodic twitches in the paralyzed limbs. The great nicety of practice in the treatment of paralysis in this way, is to keep up a certain degree of this irritation without letting it proceed to any degree of violence, and to omit it whenever the following symptoms become manifest,—head-ache, giddiness, weakness and sickness of stomach, and too violent spasmodic twitches of the limbs.

There is a great difference with respect to susceptibility of the effects of this remedy in different individuals; in some the effects speedily appear, and you are obliged to intermit its use; others will bear large doses for a considerable time, and you may push the strychnine until a grain or a grain and a half is taken in the day. I have myself given to one patient a grain every day for the space of a fortnight without any intermission. In all cases, however, it will be necessary to watch the symptoms. There is one effect of strychnine which appears to be unfavorable, and whenever it occurs you should either omit the medicine or diminish the dose. Along with or succeeding the spasms, *there is a tonic rigidity of the limbs;* when this occurs you should be cautious in the administration of strychnine. The length of time which it should be contiuued will of course vary according to circumstances, but you should be aware that it requires a considerable period of time to produce its effects. In all Dr. Bardsley's cases, and in all those treated at the Meath Hospital, it has been continued for a considerable time, certainly more than a month. It is also necessary for you to recollect that strychnine is one of those medicines which are termed accumulative; that is to say, remedies, the operation of which, after remaining latent for some time, suddenly explodes with great violence. When this occurs, the strychnine must be immediately given up, and steps taken to control its effects. One of the best things for this purpose is the carbonate of ammonia with some mild anodyne. I have seen very severe spasms from the use of this medicine. In one case these spasms were so violent as to roll the patient nearly out of bed.

It has been proposed to employ brucine as a substitute for strychnine. Of this remedy I can say but very little; I have given it but very seldom, I believe only in two cases, and in these without any sensible effect. It is much weaker than the former remedy, one-fourth of a grain of strychnine being equal to six grains of brucine. Other remedies have been proposed for the same purpose, among the rest, iodine, which has been recommended by Dr. Mansfield.

We now come to the local management of the disease, or that portion of its treatment which consists in the application of stimulants to the nerves and their origins. Local stimulation of paralytic limbs may be performed in a variety of ways; all the usual stimulant embrocations may be employed for this purpose with the best effects. I shall not take up your time in detailing the different kinds of liniments which are used on such occasions; they are universally known, and may be varied *ad infinitum*. The flesh brush, the shower-bath, either tepid or cold, occasional blisters to the spine, or along the course of the nerves, croton oil and terebinthinate friction,—all these are measures that may be employed with advantage. The use of the moxa has been also strongly recommended, and appears to be decidedly beneficial. The efficacy of all these remedies, however, seems to depend chiefly on the particular stage and nature of the disease, and hence their good effects are most apparent in those cases where the paralysis no longer depends on organic disease of the brain, but seems to be connected with that peculiar state of the nervous system which arises from a long interruption of the power of transmitting volition. It is in cases like this, that the application of the moxa has been found to produce the most favorable results. Where the lower extremities are affected, it may be applied over the sciatic nerve on the loins, or a little below and to the outer side of the politeal space over the track of the peroneal nerve. In case of paralysis of the upper extremity, you may apply it to the back of the neck, or in the neighborhood of the brachial plexus.

A gentleman, who does me the honor of attending these lectures, has related to me the particulars of a remarkable case, which I shall mention *en passant*. A young female was subject to repeated violent attacks of spasms with contraction in one of the upper extremities. She had labored under this affection for a long time, and tried various remedies without benefit. At the suggestion of this gentleman she tried cupping in the neighborhood of the shoulder and brachial plexus, and found that it produced decided relief to the symptoms. In this case it is highly probable that the disease was seated in the brachial plexus, and had no connection with the brain, for it had continued for a great length of time, (more than three years, I believe,) without any remarkable variation in its symptoms. If the spasms of the arm had been produced by irritation of the brain, she would in all probability have had paralysis long before this period; this, however, did not occur, and the probability that the disease was seated in the brachial plexus is still farther confirmed by the fact, that the spasms were relieved by local bleedings. Here we have the spasms relieved by antiphlogistic means, but in a case of atony of the same nerves, most benefit would be derived from the use of stimulants. The more completely the paralysis is of this description, the more sure will be the effects of local stimulation. You will sometimes meet with cases of paralysis from pressure on the nerves without organic disease. Thus there is a case on record, of a person who lost the use of one of his upper extremities, from having leant too long over a bench at a public meeting. I recollect the case of a man, who during a fit of intoxication fell asleep with his arm thrown over the back of chair, and awoke with perfect paralysis of the hand. Cases like these are seldom of long duration, and are much improved by the application of the moxa. I may state, however, that permanent paralysis has been induced in this way. The best way of using the moxa is, not to make a deep eschar, but to touch the parts slightly, and repeat the application frequently. In the case of paralysis of the hand, immediate relief followed the use of the moxa to the back of the wrist.

While on the subject, I may advise you always to employ the moxa in the mode first, I believe, devised by my friend and colleague Professor M'Namara. The top of the moxa is to be dipped in a strong solution of the oxymuriate of potass, which is to be allowed to dry upon it. The moxa being fixed to the part by a little gum, a drop of strong sulphuric acid will produce immediate ignition. In this way you prevent all the alarm which the patient feels at seeing a lighted candle brought to the bed-side. The same rule is to be observed when you employ electricity; the best mode of using which is to place the patient on an insulated stool, and draw sparks from, or shocks through, the affected limbs. Electricity frequently does much good in such cases; but in order to obtain decided benefit from it, you must persevere for some time in its employment. It has been lately proposed to employ the stimulus of electricity and galvanism in a different way, by transmitting it directly to the muscles of the affected limbs by means of needles, which are to be inserted into different parts of paralyzed extremities, and which are intended to act as conductors for transmitting the galvanic influence. This has been termed *electro or galvanic puncturation*, and forms an excellent mode of applying the stimulus of galvanism. I have made many experiments as to its effects, to which I shall briefly direct your attention.

The first thing to be considered is the manner of its application. The following is that which I use at the Meath Hospital:—Having procured two fine sewing needles, your first step will be to take the temper out of them; for, if you employ them in the tempered state, you will run the risk of their breaking in the flesh, and this would be very disagreeable. You can easily take the temper out of them by holding them in a candle until they become red hot, and then letting them cool gradually. The next thing is to place a head which will remain firm on the needle, and for this purpose you pass a small portion of thread through the eye, and then cover it with a bit of melted sealing wax. Having thus formed a head for the needle, you sharpen its point, and polish it by the emery pincushion, and the sharper it is the better. There is nothing more simple than to introduce the needles. You make the part of the skin tense with your finger and thumb, where you intend to introduce them, and placing the point of the needle perpendicularly on it, you press it downwards in a slanting direction, using, at the same time, a rotary motion, and thus easily pass it in; when you have pierced the skin and fascia, there is no difficulty in introducing it into the muscular fibres. The distance between the needles must be regulated according to circumstances. You then proceed to send the galvanic fluid to the part, and for this purpose, the best mode is to employ a small galvanic battery with a limited number of plates. If you have plates of from two to three square inches you will find that from fifteen to twenty of these, in a state of ordinary action, will be quite sufficient, particularly in the commencement of the treatment. It is a curious fact, that the intensity of the shock is increased to an extraordinary degree by means of the needles. A battery which, in the usual manner, would not communicate any shock, will, when used with the needles, give a violent one, and communicate such a stimulus to the nerves as will throw the whole limb into violent spasms, and cause a copious perspiration to break out over the body. I have seen very great effects from a feeble battery in this way, and it would appear that this is the result of the *direct* transmission of the galvanic influence to the muscular fibre. In most cases a perspiration is brought on, the limb convulsed, and sometimes the whole body is thrown into spasms. As an illustration of the power of the battery when used in this way, I shall mention the following case:—A patient, who was under the care of Mr. Hamilton, labored under amauroses; he was anxious to try the effect of galvanism, and with this view inserted one needle in the upper part of the back of the neck, and another over the orbit, so as to direct the trajet of the fluid across the base of the brain. He intended at first to use a small battery of twenty-five plates, but it struck him that even twenty-five might be too much. He made the experiment with three pairs of plates, and the shock being given, the patient, to his astonishment, fell back as if he had been stunned by a violent blow on the head, and remained for nearly a minute in a state of insensibility. In other cases too, where the galvanism was applied in the vicinity of the head, I have found that severe head-ache, giddiness, and even a stiffness of the muscles of the face, were produced; all showing its powerful action on the nervous centre.

Some singular circumstances, connected with this subject, were observed in the Meath Hospital. It was found that after a certain number of shocks had been communicated to the parts, when you came to withdraw the needles, there was a very remarkable difference in the ease of removing them. The needle through which the positive galvanic influence had been transmitted, was found to be strongly fastened in its situation, while that, to which the negative pole had been applied, slipped out with the greatest ease. This result was constant. In some cases, where half a dozen shocks or so have been given, the extraction of the positive needle has been only accomplished with considerable pain to the patient.

It has been suggested by a distinguished scientific friend of mine, that this results from the coagulation of albumen at the positive pole. Mr. Hamilton, however, who performed most of the operations for me, thinks that the true explanation is the paralyzing effect of the negative pole on the muscular fibre, while the positive needle is firmly grasped by the increased contraction. Farther researches are necessary on this point. Another fact connected with this subject is, that when the needles have been inserted into a large muscular mass, the positive needle is powerfully retracted, and carried as it were, into the muscles. In one case, where the needle was inserted into the lumbar muscles, in a patient laboring under sciatica, more than one-twelfth of an inch of it was drawn in at each shock ; so that, after a certain number of shocks, it passed up to the head. This is one reason for using the sealing wax head, in order to prevent the complete passing in of the needle.

With respect to our experience of the value of this mode of employing electricity or galvanism, I have to remark, that, if galvanism or electricity can be of any use to paralyzed limbs, this is one of the best modes in which it can be applied. The apparatus is simple, can be prepared in a moment, and does not depend on the state of the weather, like the ordinary electrical machines. There is another advantage, also, it is not so likely to excite alarm in the mind of the patient. We have employed it in several rheumatic and paralytic cases in the Meath Hospital, but have not as yet been able to say that decided benefit has accrued from it to the majority of the patients on whom it has been tried. This is more particularly true with respect to paralytic patients; in the rheumatic cases we have found it more beneficial. In a remarkable case, where the deltoid muscle was paralyzed and atrophied from some affection of its nerves, Mr. Hamilton tried it for a fortnight without any good effects. In a case of senile amaurosis, its effect was to produce flashes of light before the eyes, lachrymasion, and contraction of the pupil, but, after a fortnight's trial there was no improvement in the sight. We have had, however, distinct and unequivocal proofs of its value in one case of paralysis of the muscles of the face, which had all the characters of that described by Sir C. Bell, as resulting from an affection of the seventh pair of nerves. This patient had been for a long time laboring under an affection of one side of the face, and had used a variety of remedies. Those principally employed were stimulating liniments and the internal use of strychnine, from which he derived some slight benefit ; but the application of the galvanic fluid, in the way I have mentioned, was followed by decided and rapid improvement. Indeed, from the time it was first applied, the patient recovered rapidly, so that in a very short time all the deformity of face disappeared. Now the value of the application is to be estimated in this way. Here we have a case of paralysis of a local nature, and not depending upon any disease of the brain; in this case the galvano-puncturation was tried, and found to be most beneficial. The conclusion, then, as far a single case goes, is that this mode of treatment is best adapted to the form of paralysis just mentioned, in which we find an affection of some of the muscles remaining after the original disease of the brain has been removed. The same observation, I need not tell you, applies to all other remedies which are employed for the purpose of local stimulation.—*Ibid, August 9th and September 6th*, 1834.—*Ibid.*

19. *Epilepsy cured with Indigo.*—The utility of indigo as a medical agent was first pointed out by Dr. Grossheim, a Prussian physician. The results which he obtained were favorable, and Dr. Ideler was accordingly appointed to make a set of experiments on pateints affected with epilepsy. Twenty-six patients were treated, and of these six recovered perfectly; three were apparently cured, but from eight to twelve months afterwards suffered a relapse under the influence of causes which of themselves alone were sufficient to generate epilepsy. Eleven patients were essentially improved, and of the whole number treated, only six experienced no change. The effects of indigo on the animal economy are in no ways disagreeable or dangerous; at first the patients vomited frequently, without any straining or derangement of the digestive organs, after a few days this ceased, and the patient had six or eight stools a day, sometimes accompanied with slight colicky pains; after a short time the purging diminished, the matter passed assumed a fluid character, and this state continued as long as the indigo was administered, without the appetite or digestive organs being injured. The useful reaction of the remedy on the nervous system was proved by the fact, that the epileptic attacks were more severe at the commencement, and seemed to assume a higher degree of intensity, but they soon became less frequent and violent, and at length disappeared. The remedy was commonly given in the form of confection with some aromatic powder, or on other occasions simply by itself. The dose to begin with is one scruple, which may be increased to a drachm after a short time, and gradually carried to half an ounce or an ounce.—*Lancet, June 6th*, 1835, *from Berlin Medicin. Zeitung. No.* 6.—*Ibid.*

20. *Amenorrhœa cured by Turpentine Injections.*—Dr. Elliotson has successfully treated in North London Hospital, two cases of amenorrhœa, by turpentine injections. The first case was a girl aged eighteen, in whom the catamenia had disappeared about four months before, in consequence of her having caught cold. She was in good health; the pulse 72, and full; she is, however, subject to swelling of the ankles. She was immediately bled to twelve ounces; and an enema consisting of half an ounce of oil of turpentine and a pint of decoction of barley was ordered to be administered every day. On the 5th of May the catamenia appeared, and she was discharged on the 12th quite well.

The other case is that of a girl, aged sixteen, who was admitted April 14th. The catamenia had disappeared about four months previously, without the cause being known. She appeared on her admission to be in good health; her pulse 78, and rather full. Ten ounces of blood were drawn, and an injection similar to that which was employed in the last case was administered. The catamenia appeared on the 18th, four days after her admission. The enemata were discontinued, and the catamenial discharge ceased on the next day; they returned on the day following, and finally disappeared two days after that. She was bled to twelve ounces, and discharged quite well on May 12th.

Dr. Elliotson, in making some clinical remarks on these cases, said that he was induced to employ the turpentine injections in cases of suppressed menstruation from the circumstance of turpentine being considered an excellent emmenagogue when taken inetrnally. He consequently thought that it might be successfully used in the form of enema in amenorrhœa, as it was known that when it is employed for the removal of ascarides it is much more effective when administered per anum, than when given by the mouth. He had employed this remedy in the case of a lady who was partially insane in consequence of the supperssion of the catamenia. For eighteen months she had used all the established means for the removal of the disease, without effect, but the second injection of turpentine produced the menstrual discharge, and the insanity dependent on its retention was removed. It was not, however, always so effectual. He had employed it in a case now in the hospital, without success. Two injections had been given daily for some time, but the catamenia had not appeared. He thought that he had given the turpentine a fair trial in that case, and had therefore discontinued it. He had since ordered a slight injection of the liquor ammon. subcarbon. per vaginam, as recommended by an Italiau physician, but without as yet doing any good.—*Lancet*, *July* 25*th*, 1835.—*Ibid.*

21. *Breschet's Operation for the Radical Cure of Varicocele.*—The honor of increasing the domain of curative surgery, by a radical treatment of varicocele, appears to have been reserved for M. Breschet. This distinguished surgeon has, in fact, devised a plan, simple as it is ingenious, by which the most inveterate scrotal and spermatic varices are completely and expeditiously removable. I shall proceed to lay before your readers a brief account of this plan, accompanied with a case in which it was employed. I make choice of the first case in which M. Breschet operated, in order that the attendant difficulties of a first trial may not be concealed, and, at the same time, that the timid may not imagine them greater than they really were.

The patient was a servant, æt. 29, robust, and of strong constitution, who, so early as the age of fifteen, had perceived a difference in the conformation of the testicles. Some years afterwards, a tumor and varicose dilitation of the left scrotal veins became evident. The patient was frequently obliged to give up his ordinary work. He was admitted into the Hotel-Dieu, May 27th, 1833.

The diagnosis of the affection was easily established; the usual symptoms of spermatic and scrotal varicocele existed in a very marked manner. Many of the dilated veins of the scrotum equalled in size the auricular finger; and a fasciculus of vessels, united so as to form a considerable mass, originated at the cauda of the epididymis, from which point they followed the course of the cord to the external abdominal ring. The vas deferens was recognized by the characters which are peculiar to it.

The usual palliative treatment was prescribed, and its use continued during a month. The veins of the cord and scrotum, during that period, became slightly diminished in size, but the increase of volume, which followed the erect position, clearly indicated that the treatment had been, indeed, but palliative. Pressingly urged by the patient, M. Breschet thought of putting into execution a mode of treatment for a radical cure, the idea of which he had previously conceived. The principle upon which it was based, was the obliteration of the affected vein *by compression.* The compression was at first produced by small iron pincers, the pressure of which always equal, and resulted from the simple elasticity of the branches of the instrument. But the difficulty of using these pincers, their occasionally too great pressure, and the impossibility of padding their metallic surfaces, induced M. Breschet to use in their stead others so formed, as to permit the part destined to come in contact with the scrotum to be covered with soft linen, or leather, and the pressure to be graduated at will, by means of screws.

The instruments were at first applied on the veins of the scrotum, at the extremity of two of the most voluminous of them; care being taken to leave no considerable anostomosis between the two points compressed. Their presence determined slight local pain, and the trifling inflammation which followed, was combated by emollients, resolutives, and repose. On a second application, the pain was much less considerable; the graduated compression caused a thinning of the skin, and, bringing the two opposed portions of that membrane into contact, produced a dry eschar, solid, thin, and transparent, resembling parchment. Its detachment was followed by an lcueration of much less extent, and less painful than in the former instance. These ulcers were perfectly cicatrized in a few days; they produced no hæmorrhage. The vein remained full of clotted blood; this blood was gradually absorbed; no inflammation took place; and subsequently all traces of the existence of the vessel had disappeared. The different veins of the scrotum were successively treated in the same manner.

This success was, however, incomplete. The spermatic veins still remained. The application of the instrument to them was not so easily affected. They were surrounded by a thick skin containing fat, and the vas deferens was in their immediate vicinity. The small size of the nerves and arteries prevented their being drawn aside. The accidents to be apprehended were pain, violent inflammation, formation of a deep eschar, and denudation of the cord. For this new application the instruments were modified, so as to offer a larger surface to the part affected, and to present a curve, which should prevent the pressure of the fold of the skin. The vas deferens was excluded. The pain on the application was considerable, but removed by local means. The instruments were kept on seven days; one placed as near as possible to the external ring, the other towards the inferior part of the cord. They determined an inflammatory swelling of the parts comprised between the two pincers, and produced a superficial eschar of the part on which they immediately acted, combined with an adhesion of the two opposed cutaneous surfaces. The consequent ulcerations were cicatrized in less than fifteen days; before which time the cord had lost its knotty feel. There, however, still existed, at the cauda of the epididymis, a mass of vessels extending as far as the place of the last compression.

Two other pincers were applied; one immediately before the testis, which was drawn backwards, the other two inches higher up. They were kept on the same number of days as the preceding, and followed by some pain, inflammatory swelling, and eschars of the integuments.

It deserves remark, that the inflammatory symptoms, after this last operation, were more violent than before, and less promptly removed. The instrument placed above the testicle produced a perforation of the skin, and a solution of continuity resembling that produced by a seton. The inflammation and swelling were considerable; an abundant oozing of a sero-purulent matter existed during fifteen days, from the ulcer. These accidents were got under by emollient cataplasms and repose, and at the same time the varicose mass which had existed at the bottom of the scrotum, was converted into a small tumor, in which no trace of vessels was discoverable. The disappearance of the swelling soon permitted an examination of the testicle, which had been long lost amid the vast fasciculi of vessels. Its nutrition was found to be unimpaired, and its volume equal to that of the opposite side. The cure was perfect at the end of November.

The length of time occupied in the cure of this case, is by no means to be attributed to the method employed in its attainment, but to the various difficulties encountered in devising and having made the instruments employed, and to the caution necessary in the first trial of an operation of which the ultimate results could not, *a priori*, be fixed with any degree of certainty. M. Breschet has since operated in numerous cases, both in public and private practice, with perfect success, and obtained a cure in a much less time. I have myself seen two cases treated by him at the Hotel-Dieu, in which the cure was perfect in from four to five weeks.

It is exceedingly important that the compression be more than sufficient to induce an adhesive inflammation, with occasionally slight suppuration of the coats of the veins; such an effect would not only be insufficient, but might be injurious, from the propagation of the inflammation along those coats, or from the purulent infection of the blood. The pressure should be forcible enough to destroy the life of the part, but in a gradual manner. The mortification is confined to the part immediately acted on.—*London Med. Gaz., Dec.* 13*th*, 1834.—*Ibid.*

22. *Simple Fracture of the Os Femoris—Reunion—Death at the end of Two Months—Dissection.* By James Syme.—Susan Barr, aged fifty-one, was admitted on the 2d of April, in consequence of having sustained a fracture of the left thigh bone, which she stated had happened the preceding evening from being thrown down by a man who ran against her while crossing the street. The injury having been ascertained to be seated in the upper third of the bone, the limb, properly supported by splints, was placed upon a double inclined plane.

On the 15th she was suddenly seized with sickness and vomiting, and then became extremely hot and restless, with dry brown tongue and quick pulse. In three or four days these unpleasant symptoms left her, and on the 20th of May the limb was found sufficiently firm to be freed from restraint. On the 27th she had a rigor and a return of her former symptoms, which continued with progressive aggravation until the 7th of June, when she died.

The fracture had evidently been comminuted. The broken surfaces remained *quite unconnected*, a soft bloody semi-fluid substance only lying between them. In the medullary canal there had been a deposition of osseous matter in a sort of granular state, and the edges of the fracture were united by bridges of dense bone. In this case, then, the *provisional* callus of the French pathologists was nearly completed.

It is a remarkable fact in the history of pathology, that Duhamel's heory of the reunion of fractures, which was founded on an erroneous nalogy between the formation of wood and that of bone, has proved ) be much nearer the truth than that of Haller and his pupils, who ntertained correct opinions as to the formation and nourishment of one. Duhamel supposed, that, in a case of fracture, the periosteum ad its inner layer converted into bone, just as the inner layer of the ark of a graft is converted into wood, and that thus a connecting ridge was formed between the broken bones. When specimens vere shown to him of the union extending through the medullary anal, he explained the appearance by alleging, that the *internal* periosteum had suffered a similar change; and when his attention was alled to sections of old united fractures, in which a compact mass f bone occupied the seat of the fracture, he was satisfied with supposing, that the external and internal periosteum had united. Rude and crude, and ill-founded as this theory was, it approaches wonderfully near the enlightened views of Breschet and Dupuytren, who have been the first to explain satisfactorily the process by which the every-day accident of fracture is repaired. The reader is no doubt aware that the explanation formerly admitted, of an organizable substance effused from the broken bones into the space between them, and gradually hardened into bone, is quite untenable; and that the process of reunion truly consists, 1, in the formation of a capsule surrounding the fractured extremities by thickening and condensation of the neighboring tissues; 2, the deposition of bone in this capsule, and in the medullary canal; 3, the growth of bone from the surrounding osseous surfaces until the cavity is completely obliterated. The second stage is generally so far completed in from three to six weeks, that the limb regains its rigidity sufficiently to resist any moderate force, and the cure is then said to be completed; but the real cure requires at least as many months. The case that has just been related affords a striking illustration and confirmation of this process; since, if it were not for the provisional callus or bridges of new bone connecting the external edges of the fracture, the bone would still be flexible; and, in fact, one of the halves is flexible from the section having been accidentally made so as to leave the bridge more on one side than the other.—*Edinburgh Med. and Surg. Jour.*, *July*, 1835.—*Ibid.*

23. *Prolapsus Ani Cured by Nux Vomica.*—Dr. Schwartz, in an article in a late number of *Hufeland's Journal*, asserts that the nux vomica is a specific remedy for the prolapsus ani. When the disease occurs in very young children, he administers a solution of one or two grains of the extract in distilled water, and gives six or ten drops every four hours. The disease is generally cured, he asserts, on the following day. When the child is older, he gives fifteen drops, and continues the medicine eight days after the cure, to prevent a relapse. Children at the breast require very small doses, two or three drops of the solution. When the prolapsus has existed for some time, he adds a few grains of the extract of rhatany as an astringent.

24. *Notice of a Case of Urinary Calculus, in which Dr. J. Randolph successfully performed Lithotripsy.* By I. Hays, M. D.—Our number for November last, contains an account by our friend Dr. Randolph, of six cases of stone in the bladder, in all of which he successfully performed the operation of lithotripsy. Dr. Randolph informs us that since furnishing us with that communication, he has performed the same operation in six other cases, and in all with entire success.

Having been politely invited by Dr. R. to assist him in his operations upon the last of these cases, it has afforded us an opportunity of collecting some notes in relation to it, which, with the author's permission, we lay before our readers.

The subject of this case is one of our most respectable merchants, aged fifty-seven years, who dates the commencement of his complaint as far back as 1816; but it is only during the nine last years that he has had *marked* symptoms of urinary calculus. For two years his sufferings have been extreme, from spasms of the bladder, sometimes followed by profuse discharges of blood through the urethra, and from frequent and uncontrollable desire to urinate. He is often obliged to pass his urine every ten minutes, and the urgency of this call is so great that he is frequently compelled to satisfy it before he can open his dress, and he habitually wears a thick cloth to protect his clothes. His complexion and countenance are bad; and he has the appearance of one worn down by long and severe sufferings.

I saw this gentleman for the first time on the 28th of September, 1835. Dr. Randolph had previously sounded him, detected a calculus in the bladder, and had also ascertained by the introduction of bougies that the urethra was of sufficient size to admit Jacobson's *brise pierre.* The patient was not in a favorable condition for an operation, having had a chill the preceding night, and his bladder being so irritable that he was compelled to empty it a short time previous to our visit. Dr. Randolph however introduced Jacobson's instrument more particularly with a view of exploring the bladder, to ascertain its condition and the size of the stone, than with an expectation of being able to proceed further with the operation; but the stone being readily caught and the patient suffering less than was anticipated, the calculus was twice caught and readily crushed, before the instrument was withdrawn. It was not thought adviseable to do more at this time, in the existing condition of the patient.

I again visited this patient on the 1st of October. He had passed since the operation, a considerable number of fragments of the calculus. These consisted of small granules cemeted together, and presenting the appearance of a mulberry calculus. The patient thinks himself benefited by the operation. Dr. R. introduced Jacobson's instrument; this part of the operation occupied but five seconds; in five seconds more a calculus was seized. The stone was caught and crushed four times, the instrument freed from the adhering fragments and withdrawn, and the whole operation completed in a few seconds less than five minutes.

The patient was seen by me for the third time on the 15th of October. His general appearance was much improved, and he expressed himself as greatly relieved. He had passed a number of fragments since the last operation, and now thinks that there is one remaining too large to pass the urethra, as he states that he feels it occasionally entering the neck of the bladder, which causes him pain. Dr. R. introduced Jacobson's instrument, caught several times some fragments and crushed them.

At the request of Dr. Randolph, I this day, (Oct. 25th,) sounded the patient and could not detect any fragments in the bladder. The exploration did not occasion the patient any pain. The appearance of the patient is now greatly improved, his complexion and countenance are good, his spirits excellent. He says that he is entirely well, can retain his urine four or five hours, and walk as actively as any one. It will be perceived that this case was completed by three operations, and in the short period of but little more than two weeks. From the quantity of fragments collected, we should suppose the stone was of the size of a small walnut.

We questioned this patient after each operation and again to-day respecting the pain he suffered from them. His answers have always been the same, that the pain "from the operation was nothing compared to what he often suffered from the spasms of the bladder." Indeed he never uttered a groan, or desired that the operation might be desisted from. He now says that the principal pain he experienced, was "during the working of the instrument," that as soon as this was stopped or the instrument withdrawn, all pain ceased, leaving only a slight smarting or soreness at the end of the penis. After each operation he experienced marked relief. We were particular in our inquiries respecting the pain suffered from the operation, and have stated the answers as we received them, nearly in the words of the patient, because, much difference of opinion exists on this point. It is one, however, not readily settled, inasmuch as we have no positive measure of pain, and moreover, its degree must vary in each case, according to the condition of the bladder, the sensibility of the patient to pain, and finally, it must depend in no small measure upon the kind of instrument employed and the skill of the operator. We must not conclude these imperfect notes without expressing the admiration we experienced at the neatness and skill displayed by Dr. R. in his manipulation, upon which in no small measure must depend his great success.

